# Replacing Soybean Meal with Ultrafine Pulverized Mulberry Leaf (Combined with Multi-Enzyme Preparations) Reduces Fat Deposition and Alleviates Oxidative Stress in Grass Carp via Gut–Liver Axis Regulation

**DOI:** 10.3390/antiox15101350

**Published:** 2026-10-19

**Authors:** Haotian Yuan, Ling Long, Yelin Zeng, Long Huang, Yaoyu Chen, Yongsheng Wang

**Affiliations:** 1 Agricultural Genomics Institute at Shenzhen, Chinese Academy of Agricultural Sciences, Shenzhen 518000, China; yhtian0125@163.com (H.Y.); zengyelin@caas.cn (Y.Z.); 2 Kunpeng Institute of Modern Agriculture at Foshan, Foshan 528225, China; m13866100086@163.com; 3 School of Bioengineering, Northwest Minzu University, Lanzhou 730030, China; 278061354@xbmu.edu.cn; 4 Foshan Institute of Agricultural Sciences, Foshan 528000, China; huanglong@163.com; 5 School of Life Sciences, South China Normal University, Guangzhou 528225, China

**Keywords:** antioxidant capacity, lipid metabolism, multi-omics, phenotypic

## Abstract

Grass carp are prone to excessive fat deposition in intensive aquaculture, which impairs their health and reduces economic returns. Here, we investigated the effects of replacing soybean meal with ultrafine pulverized mulberry leaves combined with multi-enzyme preparations on lipid metabolism in grass carp using phenotypic and multi-omics approaches. A total of 480 grass carp (8.45 ± 0.17 g) were fed a basal diet and two experimental diets (6% and 12%) containing mulberry leaf for 60 d. Replacing soybean meal with ultrafine pulverized mulberry leaf with multi-enzyme preparations reduced serum triglyceride, total cholesterol, high-density lipoprotein cholesterol, low-density lipoprotein cholesterol, and dorsal muscle ether extract levels, downregulated hepatic glycolysis (*gk*, *pfk*, *pk*) and lipogenesis (*fas* and *acc*) genes and increased intestinal lipase activity. Intestinal omics identified the lysine degradation pathway as a key pathway that regulates lipid metabolism. Reduced levels of multiple differential metabolites and enhanced microbial metabolic functions are closely associated with inhibition of lipid metabolism in grass carp. The reduction in fat deposition, particularly polyunsaturated fatty acids, the downregulation of antioxidant capacity, as indicated by decreased glutathione peroxidase and superoxide dismutase activities and reduced total antioxidant capacity, along with a decrease in malondialdehyde levels, suggests that the lipid-lowering effects could alleviate oxidative stress. Replacing soybean meal with ultrafine pulverized mulberry leaves combined with multi-enzyme preparations reduced lipid deposition and alleviated oxidative stress in grass carp by regulating the gut–liver axis. When replacing soybean meal, a 12% proportion of ultrafine pulverized mulberry leaves combined with multi-enzyme preparations in the diet is recommended.

## 1. Introduction

Diet can influence the metabolism of grass carp (*Ctenopharyngodon idella*) by affecting the composition, interspecific interactions, and gut microbiota functions [1]. In modern aquaculture, high-energy compound feeds are used to spare protein. However, long-term feeding of high-energy diets leads to excessive fat accumulation, which is an important factor that reduces fish tolerance to and utilization efficiency of high-energy diets. This limits the application of high-energy feeds in the aquaculture industry [2,3]. Lipids are particularly prone to reactive oxygen species (ROS) attack, which leads to lipid peroxidation, cell membrane damage, and toxic byproducts that affect DNA, proteins, and low-density lipoproteins [4]. Lipid metabolism disorders induced by high-energy diets can interact with oxidative stress [3], amplifying impacts on grass carp. Therefore, it is important to improve lipid metabolism.

Mulberry leaves have a high nutritional value and are rich in bioactive substances. This makes them an unconventional feed resource with high development potential and use value. The safety of mulberry leaves as animal feed has been verified [5], laying a foundation for their application in aquaculture. Mulberry leaves have considerable lipid metabolism-regulating abilities in livestock [6,7,8,9] and aquatic animals [10,11,12,13]. The strategic use of mulberry leaves in feed may reduce lipid levels while partially replacing conventional protein resources.

Physical modification technologies, such as ultrafine pulverization, can alter the physicochemical properties of feed materials and promote the release of bioactive components. They have been widely applied to improve the use efficiency and bioactivity of feed ingredients [14,15,16,17,18,19]. This enhances the regulatory effects of active substances on lipid metabolism, reducing lipid accumulation in animals [15,20]. Ultrafine pulverization may have similar beneficial effects as mulberry leaf treatment; that is, it enhances the release and bioavailability of key lipid-lowering active components, such as flavonoids, in mulberry leaves. This can more effectively regulate grass carp lipid metabolism. However, this may also lead to an increased release of antinutritional factors, such as phytic acid. Exogenous enzyme preparations can reduce the levels of antinutritional factors in feed ingredients, improve use efficiency, and increase the beneficial effects of mulberry leaves as a feed supplement [21]. Therefore, combining ultrafine pulverized mulberry leaves with multi-enzyme preparations may enhance lipid-lowering efficacy while reducing potential negative effects.

Although published studies have provided an important basis for understanding the lipid-lowering effects of mulberry leaves [6,7,8,11,12,13], most have focused on terminal organs and serum parameters. Here, we hypothesized that replacing soybean meal with ultrafine pulverized mulberry leaves, combined with multi-enzyme preparations, would regulate hepatic lipid metabolism gene expression and improve systemic lipid homeostasis by reshaping the gut microbiota structure and altering metabolite composition. Therefore, we investigated the effects and regulatory mechanisms of replacing soybean meal with ultrafine pulverized mulberry leaves combined with multi-enzyme preparations on lipid metabolism in grass carp from multiple perspectives. This included the gut microbiota, metabolites, genes, and biochemical indicators. We also evaluated the dose–response relationship. In this study, we aimed to investigate the lipid-lowering effects of replacing soybean meal with ultrafine pulverized mulberry leaf combined with multi-enzyme preparations in grass carp and its potential association with oxidative stress, providing a theoretical basis for its application as a functional feed additive.

## 2. Materials and Methods

### 2.1. Experimental Materials

Dried mulberry leaves (17.80% crude protein (CP), 4.46% ether extract (EE), 9.44% CF, 12.30% ash, air-dried base) were purchased from Shichao Seedling Planting Farmers’ Professional Cooperative in Suiyang District, Shangqiu City, Henan Province. Phytase (50,000 IU/g), pectinase (15,000 IU/g), and complex enzymes (cellulase, 2000 IU/g, xylanase, 4500 IU/g, and glucanase, 6000 IU/g) were purchased from DSM Co., Ltd. (Shanghai, China).

### 2.2. Experimental Diets

Dried mulberry leaves were pulverized and sieved through a 325-mesh sieve. The other ingredients were pulverized and sieved through a 60-mesh sieve. The three diets tested were a basal diet (CK) containing 0% mulberry leaf, and two treatment diets supplemented with 6% and 12% ultrafine pulverized mulberry leaf. Hereafter, the 6% and 12% groups are collectively referred to as the mulberry leaf groups. The total proportion of soybean meal and ultrafine pulverized mulberry leaf powder in the feed was constant, with the ratios of replacement of soybean meal by ultrafine pulverized mulberry leaf being 0%, 19.61%, and 39.22%. The two treatment diets were supplemented with equal amounts of phytase (0.02 g/kg, 1000 IU/kg), pectinase (0.08 g/kg, 1200 IU/kg), and complex enzyme (0.90 g/kg, cellulase 1800 IU/kg, xylanase 4050 IU/kg, glucanase 5400 IU/kg). Micro-ingredients were added using the stepwise expansion method. The feed ingredients were uniformly mixed, stirred with an appropriate amount of water in a blender, and pelleted using a DGP-60C feed extruder (Xingtai Juchi Machinery Manufacturing Co., Ltd., Xingtai, China) with a particle size of 2.00 mm. After natural cooling and air-drying, the pellets were stored in a −20 °C refrigerator for later use. The feed ingredients used were from the same source and batch and were prepared and packaged simultaneously and under the same conditions after determining the ingredient composition. All the feed formulations met the nutritional requirements of grass carp (formula feed for grass carp, *C. idellus*, GB/T 36205-2018) [22]. The feed composition and nutrient levels are listed in Table 1.

### 2.3. Experimental Design and Husbandry Management

Grass carp were purchased from the Fuyuan Fry Farm in Guangzhou City, Guangdong Province. The feeding trial was conducted in ponds at the Modern Fishery Science and Technology Park in Foshan City using net cages (1.35 × 1.35 × 2.45 m). After 14 d of acclimation feeding and 24 h of fasting, a total of 480 grass carp (8.45 ± 0.17 g) were randomly assigned to three groups. Each treatment comprised four replicates with 40 fish per replicate. Fish were reared for 60 consecutive days and fed twice daily at 8:30–9:00 and 17:00–17:30. The initial feeding rate was determined based on the feeding condition during the acclimation period and maintained at 2.00–5.00% of body weight. The amount of feed offered was gradually increased according to the actual feed intake (FI), with real-time observations of satiation. During the trial, the culture water was aerated 24 h a day, with the water temperature maintained at 27.00 ± 2.00 °C, dissolved oxygen > 5.00 mg/L, pH 7.50–8.50, and ammonia nitrogen < 0.20 mg/L. The photoperiod followed the natural light cycle of Foshan, Guangdong, China.

### 2.4. Experimental Data Collection

The initial body weight of each fish was recorded before the trial, and the FI, number of dead fish, and body weight were recorded during the rearing period. When the rearing period ended, the fish were fasted for 24 h and the final body weight was measured to obtain the average final body weight. In each replicate, six fish with body weights close to the average were anesthetized (MS-222 solution, 100 mg/L) and blood was collected from the caudal vein using a 1 mL syringe. Blood samples of approximately 0.5 mL were placed in 1.5-mL centrifuge tubes, allowed to stand at room temperature for 30 min, and centrifuged at 4 °C and 3000× *g* for 10 min to separate the serum. Serum from each replicate was pooled and stored at −80 °C until analysis. Three of the bled fish were used for tissue sampling: dorsal muscle was collected, weighed, pooled, and stored at −20 °C; liver was collected and weighed, rinsed with 0.1% DEPC water, pooled, snap-frozen in liquid nitrogen, and stored at −80 °C. Mid-hindgut samples were collected, pooled, snap-frozen in liquid nitrogen, and stored at −80 °C until analysis. Each treatment comprised four replicates.

### 2.5. Calculation of Growth and Feeding Parameters

The formulas for growth and feeding-related parameters are as follows:

FI (g/fish) = F/[(N_t_ + N_0_)/2] (1)

Weight gain rate (WGR, %) = 100 × (W_t_ − W_0_)/W_0_ (2)

Specific growth rate (SGR, %/d) = 100 × (lnW_t_ − lnW_0_)/t (3)

Feed conversion ratio (FCR) = F/(W_t_ × W_0_) (4)

Survival rate (SR, %) = 100 × N_t_/N_0_ (5)

Hepatosomatic index (HSI, %) = 100 × W_h_/W_t_ (6)

where t = days of culture; W_t_ = final body weight of fish (g); W_0_ = initial body weight of fish (g); F = total feed offered (g); N_t_ = final number of fish; N_0_ = initial number of fish; and W_h_ = liver weight (g).

### 2.6. Serum Index Determinations

Serum indices included lipid metabolism and antioxidant parameters. Total cholesterol (TC), triglyceride (TG), high-density lipoprotein cholesterol (HDL-C), and low-density lipoprotein cholesterol (LDL-C) levels were determined using an automatic biochemical analyzer (URIT-8021A, URIT, Guilin, China). The kits were purchased from Guilin URIT Medical Electronic Co., Ltd. (catalog numbers: U82785040, U82885040, U82985045, and U83085045). The assays were performed according to the manufacturer’s instructions. Total antioxidant capacity (T-AOC), malondialdehyde (MDA) levels, and the activity levels of superoxide dismutase (SOD) and glutathione peroxidase (GSH-Px) were determined using micro-methods. Kits with catalog numbers UPLC-W-A504, UPLC-W-A401, UPLC-W-A500-WST-8, and UPLC-W-A202 were purchased from Shanghai UPLC Detection Technology Co., Ltd., Shanghai, China. The assays were performed according to the manufacturer’s instructions.

### 2.7. Determination of Muscle Quality and Fatty Acid Composition

Muscle quality indices included pH, meat color, shear force, cooking loss, and centrifugal loss (CL). Muscle composition indices included CP, EE, ash, and fatty acid composition.

(1) The pH was measured using a pH-STAR carcass meat pH direct meter (Matthäus GmbH; Eckelsheim, Germany). Each sample was measured three to five times, and the average was calculated after removing outliers.
(2) Meat color was measured using an OPTO-STAR CPU carcass meat color meter (Matthäus GmbH). The Opto-Wert (optical value) range was 0–100%. Meat quality satisfaction and grading: Opto-Wert > 63 = excellent; 53 ≤ Opto-Wert < 63 = good; Opto-Wert ≤ 53 = poor.
(3) Shear force: The samples were cut into strips along the direction of the muscle fibers. Muscle shear force was measured perpendicular to the muscle fibers using a C-LM3 digital tenderness meter (Beijing Bulader Technology Development Co., Ltd., Beijing, China). The prepared meat strips were placed on the shear table of the instrument, and the shear operation was performed by pressing the start button. The shear force (N) displayed on the screen was recorded. Each sample was sheared three to five times, and the average value was calculated after removing any outliers.
(4) Cooking loss: Approximately 5 g of the muscle was placed in boiling water and boiled for 5 min. After drying the muscle with filter paper, the weights were recorded. The formula used is as follows:

Cooking percentage (%) = (cooked muscle weight/initial muscle weight) × 100

(5) CL: Moisture and blood stains on the dorsal muscle were blotted with absorbent paper and the muscle was weighed. The muscle was placed in a centrifuge tube with an appropriate amount of absorbent paper at the bottom and centrifuged at 4000× *g* for 20 min at 4 °C. After blotting with absorbent paper to remove moisture, muscle samples were reweighed. The calculation is as follows:

CL (%) = 100 × (pre-centrifugation muscle weight − post-centrifugation muscle weight)/pre-centrifugation muscle weight.

(6) CP was determined using the Kjeldahl method (GB 5009.5-2016) [23], ash using the ignition method (GB 5009.4-2016) [24], and EE using the Soxhlet extraction method (GB 5009.6-2016) [25].
(7) Fatty acids were determined by gas chromatography, and the analysis was performed by Shanghai UPLC Detection Technology Co., Ltd. The detection method is described in the Supplementary Material, that is, the analysis workflow for muscle fatty acids, gut microbiota, and intestinal non-targeted metabolomics.

### 2.8. Analysis of Gene Expression Abundance Related to Hepatic Glycolipid Metabolism

Gene primers were synthesized by Sangon Biotech Co., Ltd. (Shanghai, China), and the primer sequences are listed in Table 2. The steps for gene extraction and relative quantification were as follows. Total RNA was extracted from the liver using an Animal Tissue Total RNA Kit (spin column type DP431; TIANGEN, Beijing, China). RNA integrity was verified by agarose gel electrophoresis. The RNA concentration was measured using an Equalbit RNA HS Assay Kit (Vazyme Biotech Co., Ltd., Nanjing, China) and a Qubit 4 fluorometer (Thermo Fisher Scientific, Waltham, MA, USA). RNA purity requirements: optical density (OD) 260/280 = 1.9–2.1, OD 260/230 ≥ 1.9.

Reverse transcription was performed according to the instructions for the HiScript III 1st Strand cDNA Synthesis Kit (+gDNA wiper) (R312-01; Vazyme Biotech Co., Ltd.). The reverse-transcribed cDNA was stored at −20 °C until use. Quantitative real-time PCR (qPCR) was performed using the Taq Pro Universal SYBR qPCR Master Mix (Q712-02; Vazyme Biotech Co., Ltd.). Each sample was run in triplicate with technical replicates. β-actin was used as the internal reference gene, and the relative mRNA expression level was calculated using the 2^−ΔΔCt^ method.

### 2.9. Analysis of Intestinal Digestive Enzymes, Microbiome and Non-Targeted Metabolome

The kit for detecting intestinal tissue lipase activity (UPLC-W-B402) was purchased from Shanghai UPLC Detection Technology Co., Ltd. The assay was performed in strict accordance with the manufacturer’s instructions. The intestinal microbiome and non-targeted metabolome analyses were performed by Shanghai Personalbio Technology Co., Ltd., Shanghai, China. The details of the methods are provided in the Supplementary Material, including the analysis workflow for muscle fatty acids, gut microbiota, and intestinal non-targeted metabolomics.

### 2.10. Data Statistics and Analysis

Experimental results were reported to two decimal places, and statistical data were expressed as mean ± standard error of the mean (Mean ± SEM). Statistical significance was evaluated using one-way analysis of variance (ANOVA) followed by Duncan’s multiple range test. Differences were considered statistically significant at *p* < 0.05.

## 3. Results

### 3.1. Determination of Growth Performance, Serum Indicators, and Intestinal Lipase Activity

The growth performance is presented in Table 3. No significant differences were observed in the initial body weight, final body weight, FI, WGR, SGR, FCR, or SR among the groups (*p* > 0.05). However, the HSI was significantly lower in the mulberry leaf groups (*p* < 0.05). Serum indicators and intestinal lipase activities of grass carp are shown in Table 4. With increasing substitution levels of ultrafine pulverized mulberry leaves combined with multi-enzyme preparations, serum TG levels decreased significantly. Meanwhile, lipase activity increased significantly (*p* < 0.05). Serum TC, HDL-C, LDL-C levels, GSH-Px, and SOD activities in the 6% and 12% groups were significantly lower than those in the CK group (*p* < 0.05). The T-AOC of grass carp was significantly lower in the 12% group (*p* < 0.05), with no significant differences between the CK and 6% groups (*p* > 0.05). Serum MDA levels significantly increased in the 6% group and significantly decreased in the 12% group (*p* < 0.05).

### 3.2. Analysis of Conventional Nutrient Components and Fatty Acid Composition in Dorsal Muscle

The physicochemical properties, conventional nutrient components, and fatty acid composition of the dorsal muscles of grass carp are shown in Table 5. No significant difference in cooking loss was observed between groups (*p* > 0.05). Muscle pH in the 6% and 12% groups was higher than that in the CK group, and CL was significantly lower than that in the CK group (*p* < 0.05). The Opto-Wert in the 12% group was significantly lower than that in the 6% group (*p* < 0.05). However, both groups showed no significant differences compared with the CK group. Muscle shear force in the 12% group was significantly lower than that in the CK and 6% groups (*p* < 0.05). The CP content in the dorsal muscle of grass carp was significantly lower in the 12% group than in the 6% group (*p* < 0.05). EE content decreased significantly with increasing substitution levels of ultrafine pulverized mulberry leaves combined with multi-enzyme preparations (*p* < 0.05). No significant differences in ash content were found between the groups (*p* > 0.05). For fatty acid composition (Table 6), compared with the CK group, the contents of unsaturated fatty acids (UFA), polyunsaturated fatty acids (PUFA), ω-3 PUFA, and ω-6 PUFA decreased in the 6% group but increased in the 12% group. Meanwhile, monounsaturated fatty acid (MUFA) showed the opposite trend (*p* < 0.05). The 6% group had the lowest saturated fatty acid content (*p* < 0.05).

### 3.3. Analysis of Gene Expression Abundance Related to Hepatic Glycolipid Metabolism

**Figure 1:**
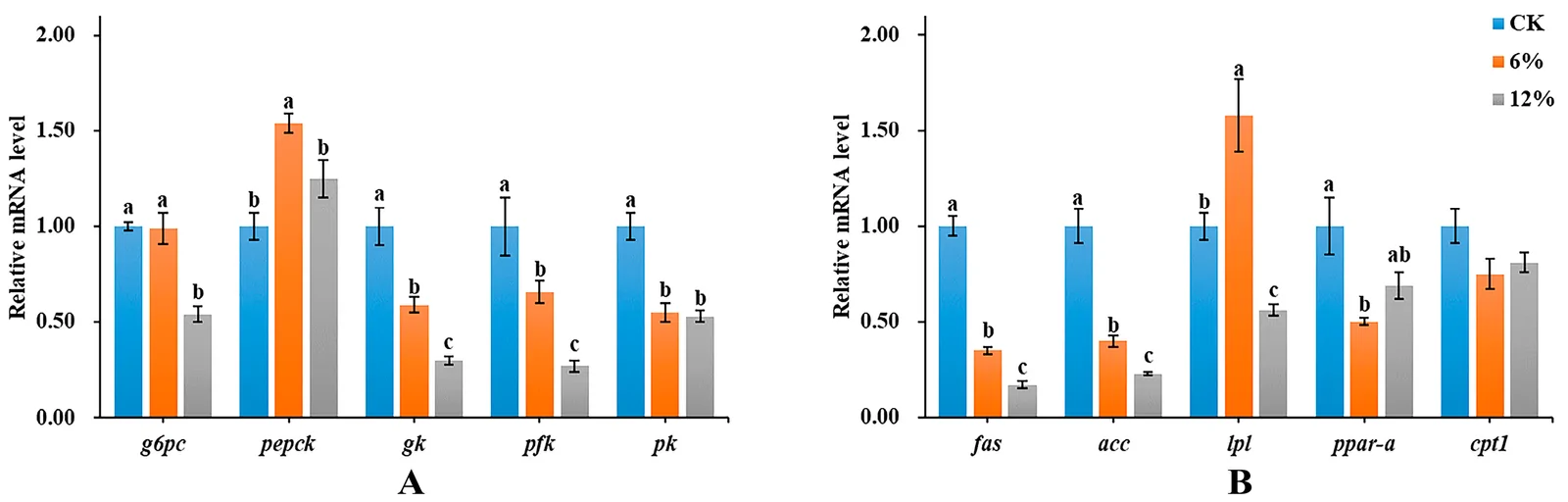
Hepatic gene expression (*n* = 4). (**A**) Genes involved in glucose metabolism. (**B**) Genes involved in lipid metabolism. The *x*-axis represents gene names, the *y*-axis represents relative expression levels, different colors correspond to different groups, and different lowercase letters indicate significant differences among groups (*p* < 0.05). *g6pc*, *glucose-6-phosphatase*; *pepck*, *phosphoenolpyruvate carboxykinase*; *gk*, *glucokinase*; *pfk*, *phosphofructokinase*; *pk*, *pyruvate kinase*; *fas*, *fatty acid synthase*; *acc*, *acetyl-CoA carboxylase*; *lpl*, *lipoprotein lipase*; *ppar-α*, *peroxisome proliferator-activated receptor alpha*; *cpt1*, *carnitine palmitoyltransferase 1*.

Given that hepatic metabolism is a key factor affecting body fat deposition, we sought to identify the genes related to hepatic glycolipid metabolism. The relative expression levels of glycolytic genes (*gk*, *pfk*, *pk*) and lipogenic genes (*fas* and *acc*) were significantly reduced in the treatment groups. The expression levels of all indices, except *pk*, decreased significantly with increasing substitution levels of ultrafine pulverized mulberry leaves combined with multi-enzyme preparations (*p* < 0.05; Figure 1). In terms of gluconeogenesis genes, the expression level of *g6pc* in the 12% group was significantly lower than that in the CK and 6% groups (*p* < 0.05) and the level of *pepck* in the 6% group was significantly higher than in the CK and 12% groups (*p* < 0.05; Figure 1A). For lipolysis genes, compared with the CK group, the expression level of *lpl* was significantly increased in the 6% group (*p* < 0.05) and significantly decreased in the 12% group (*p* < 0.05). Meanwhile, the expression level of *ppar-α* was significantly reduced in the 6% group (*p* < 0.05). No significant difference in *cpt1* expression was observed among the groups (*p* > 0.05) (Figure 1B).

### 3.4. Differential Metabolite Screening

**Figure 2:**
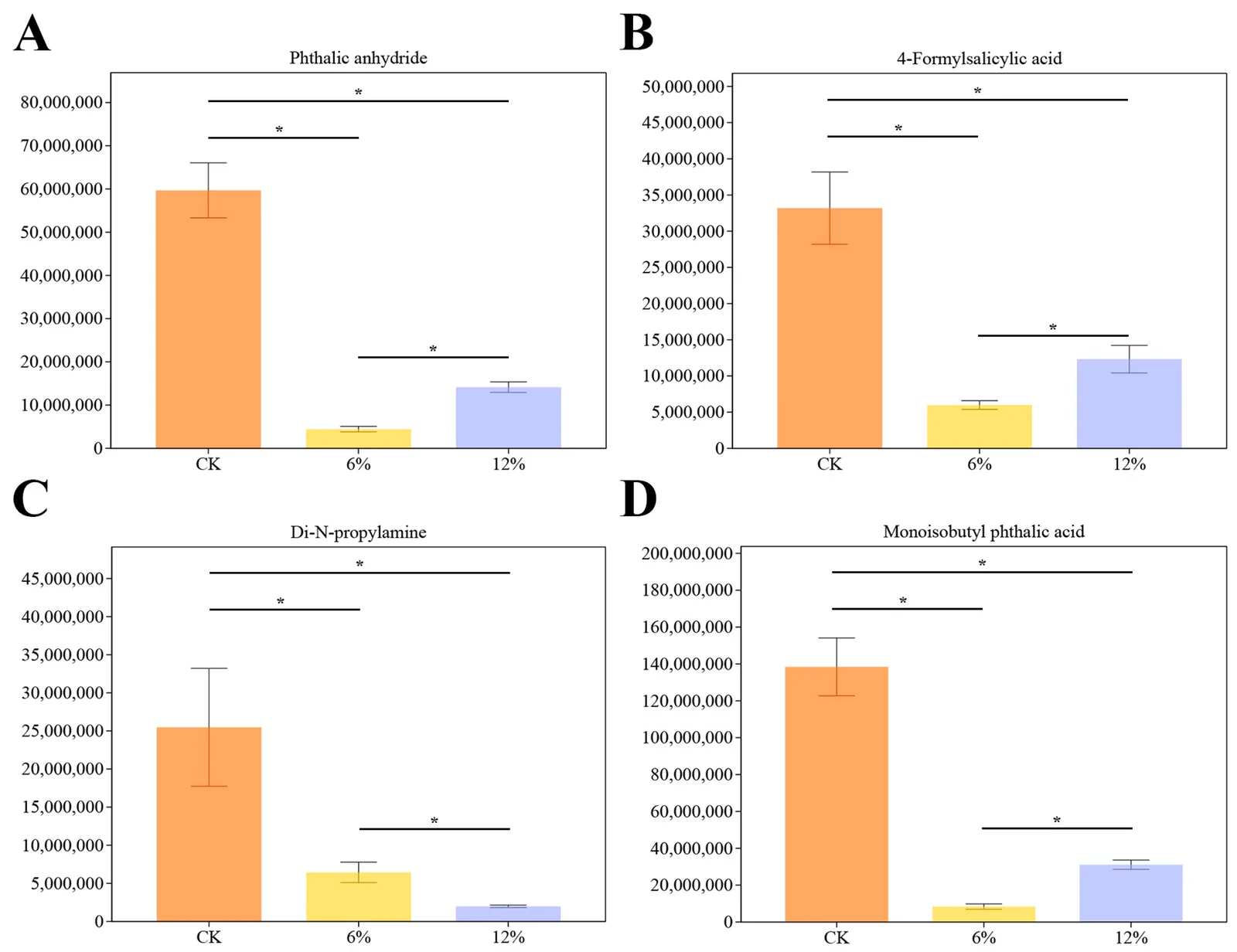
Analysis of differential metabolite expression levels (*n* = 4). The *x*-axis represents different groups, and the *y*-axis represents the signal value range of metabolites. * indicates significant differences between two groups, *p* < 0.05.

Quality evaluation of the experimental data indicated that they were suitable for subsequent analyses (Supplementary Figure S1). Partial least squares discriminant analysis showed that the samples from each group were separated under positive and negative ion modes, with significant differences in the metabolites among the groups (Supplementary Figure S2). In all the samples, 153 (92 upregulated and 61 downregulated), 103 (53 upregulated and 50 downregulated), and 44 (23 upregulated and 21 downregulated) significantly different metabolites were identified in the CK vs. 12%, CK vs. 6%, and 6% vs. 12% groups, respectively (Figures S3 and S4). The top four substances with the highest importance in the CK vs. 6% vs. 12% group model were selected as biomarker metabolites using random forest plots. These substances were also differentially metabolized in the CK vs. 12%, CK vs. 6%, and 6% vs. 12% groups (Supplementary Figure S5). Compared with the CK group, the abundances of phthalic anhydride, 4-formylsalicylic acid, di-N-propylamine, and monoisobutyl phthalic acid were significantly reduced in the 6% and 12% groups. The level of di-N-propylamine was significantly lower in the 12% group than in the 6% group. However, the levels of all other substances were higher in the 12% group (Figure 2). Based on the metabolite information, a correlation analysis was performed for the top 50 metabolites (Figure S6). In the comparison between the CK and 6% groups, phthalic anhydride, 4-formylsalicylic acid, and monoisobutyl phthalic acid were positively correlated with each other. In the comparison between the CK and 12% groups, phthalic anhydride, di-N-propylamine, and monoisobutyl phthalic acid were positively correlated with each other. In the comparison between the 6% and 12% groups, di-N-propylamine showed a negative correlation with phthalic anhydride, no significant correlation with 4-formylsalicylic acid, and a negative correlation with monoisobutyl phthalic acid. Meanwhile, the other pairs were positively correlated. In this model, phthalic anhydride was positively correlated with monoisobutyl phthalic acid.

### 3.5. Differential Metabolite Biological Function Analysis

**Figure 3:**
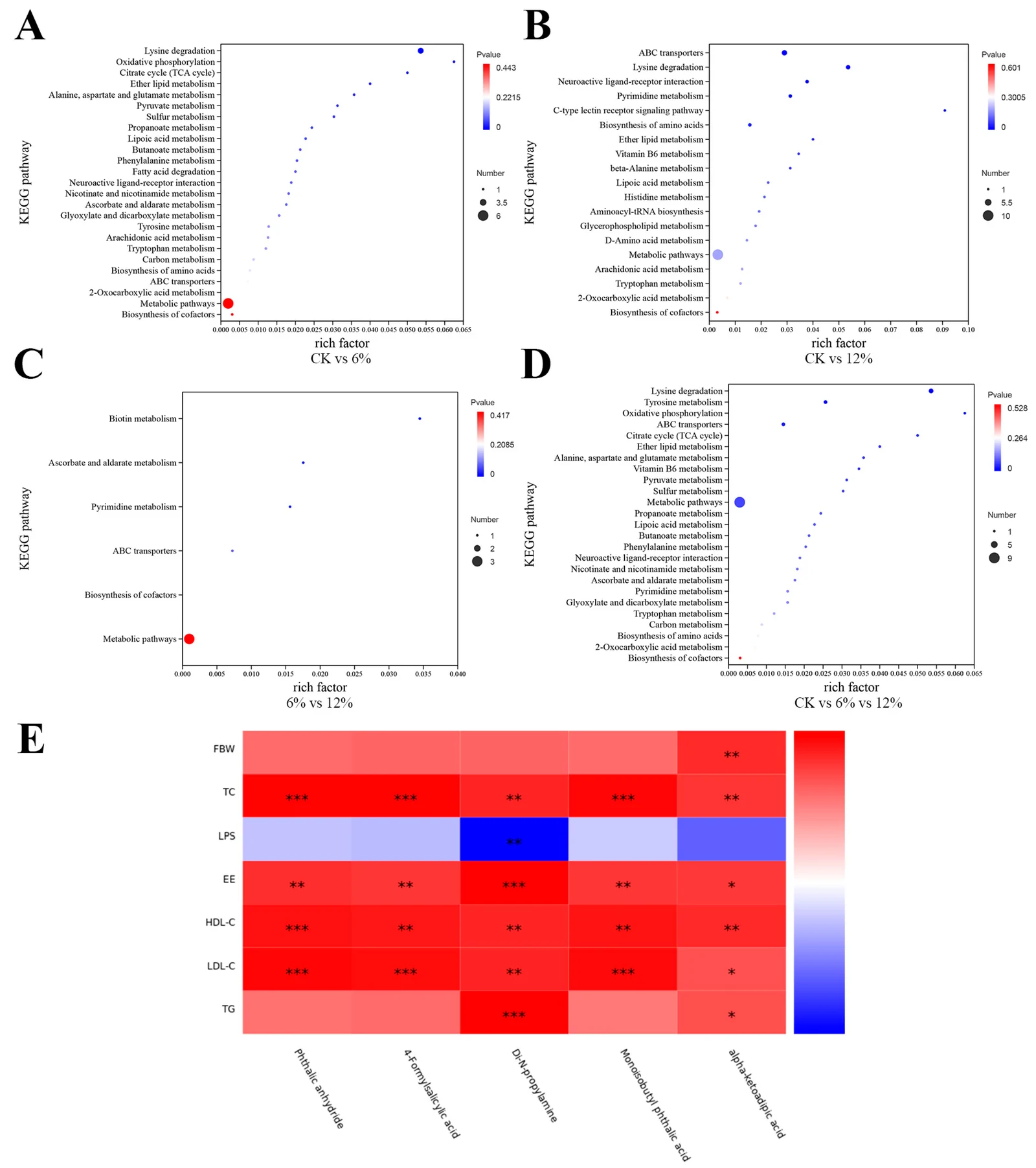
Biological function analysis of differential metabolites (*n* = 4). (**A**–**D**) Factor plots of differential metabolite enrichment analysis. (**E**) Interaction correlation heatmap between differential metabolites and lipid metabolism phenotypic indices. Blue represents negative correlation, red represents positive correlation. * indicates significant differences between two groups, * *p* < 0.05, ** *p* < 0.01, *** *p* < 0.001.

Kyoto Encyclopedia of Genes and Genomes (KEGG) enrichment analysis showed that differentially expressed metabolites were significantly enriched in the lysine degradation pathway. In CK vs. 6%, succinic acid, alpha-ketoadipic acid, and glutaric acid were downregulated. In CK vs. 12%, deoxycarnitine was upregulated, whereas alpha-ketoadipic acid and (3S)-3,6-diaminohexanoate were downregulated. In CK vs. 6% vs. 12%, the differential metabolites were succinic acid, alpha-ketoadipic acid, and (3S)-3,6-diaminohexanoate (Figure 3A–D, Supplementary Table S1). Alpha-ketoadipic acid was a common differential metabolite in the lysine degradation pathway. Further correlation analysis showed that final body weight was significantly positively correlated with alpha-ketoadipic acid. TG was significantly positively correlated with di-N-propylamine and TC, EE, HDL-C, and LDL-C were significantly positively correlated with phthalic anhydride, 4-formylsalicylic acid, di-N-propylamine, monoisobutyl phthalic acid, and alpha-ketoadipic acid. Lipase activity was significantly negatively correlated with di-N-propylamine (*p* < 0.05, Figure 3E).

### 3.6. Analysis of Intestinal Microbial Alpha and Beta Diversity

**Figure 4:**
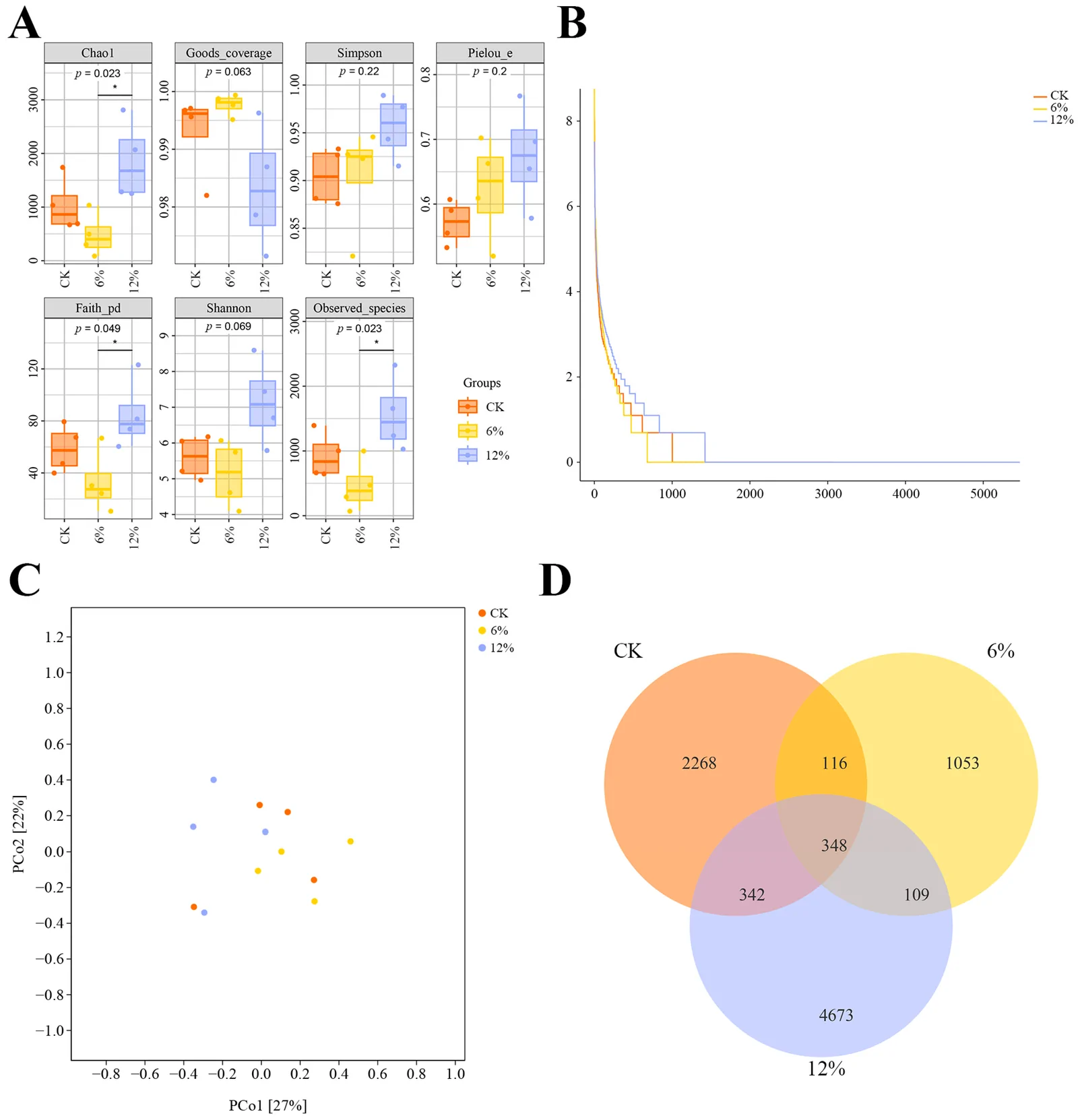
Microbiome analysis (*n* = 4). (**A**) Alpha diversity indices. Numbers under diversity index labels are *p*-values from the Kruskal–Wallis test. * indicates significant differences between two groups, *p* < 0.05. (**B**) Rank–abundance curve. (**C**) PCoA analysis. (**D**) Venn diagram of ASVs/OTUs.

Alpha diversity analysis showed significant differences in microbial richness (Chao1, Observed species) and Faith’s phylogenetic diversity (PD) among the groups, with the highest values in the 12% group and the lowest in the 6% group (Figure 4A). The steep and narrow span of the rank–abundance curve indicated low species richness and evenness, that is, a simple community structure dominated by a few dominant bacteria (Figure 4B). The beta diversity results showed no significant differences in the microbial community structure among the groups (Figure 4C). Venn diagrams were used to investigate the number of shared and unique species among groups. The number of shared amplicon sequence variants (ASVs) or operational taxonomic units (OTUs) among all the groups was 348. The numbers of unique ASVs or OTUs in the CK, 6%, and 12% groups were 2268, 1053, and 4673, respectively (Figure 4D).

### 3.7. Analysis of Abundance of Intestinal Dominant Microbiota

**Figure 5:**
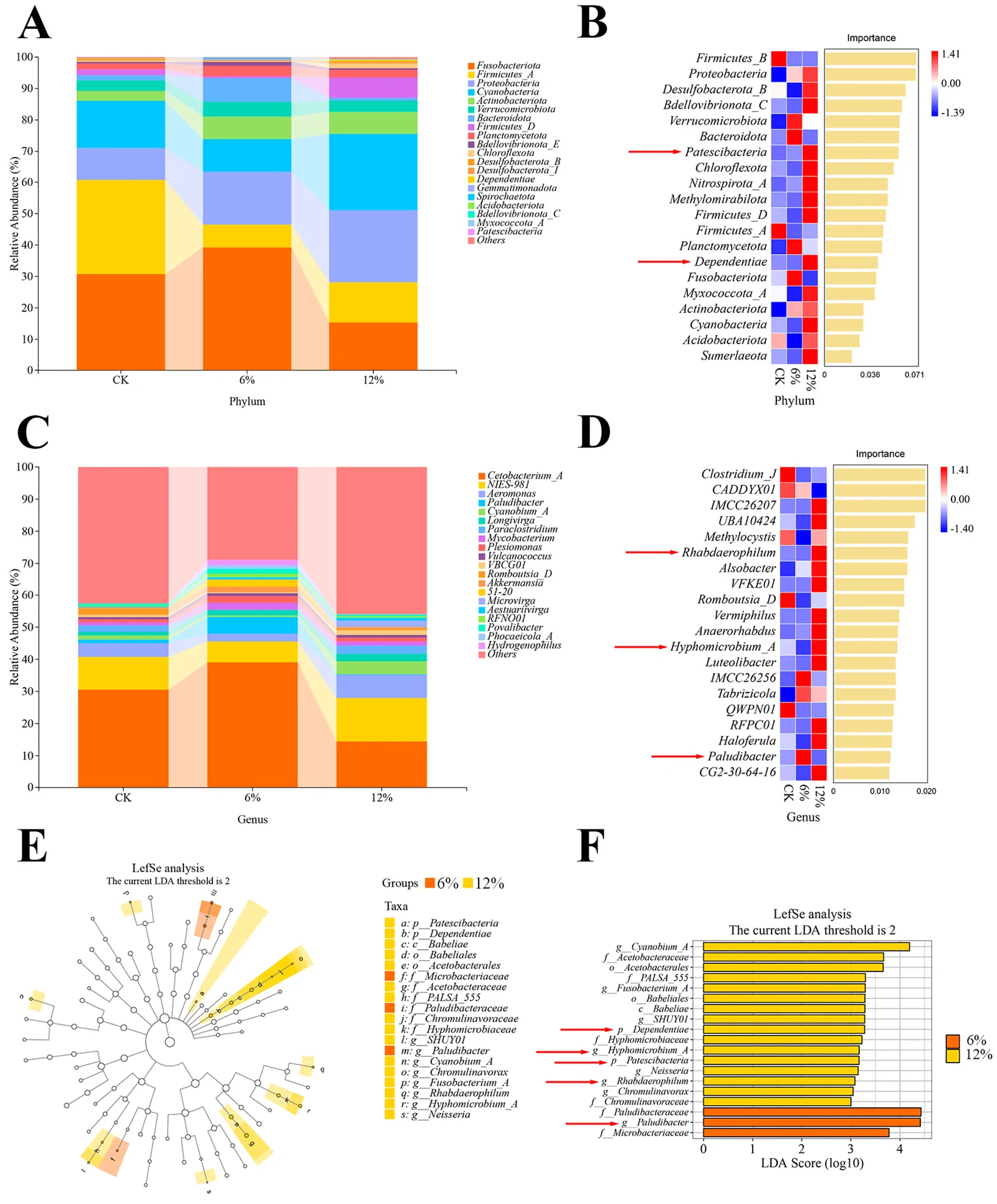
Screening of dominant and biomarker microbes (*n* = 4). (**A**,**C**) Species composition plots at phylum/genus level. (**B**,**D**) Random forest analysis at phylum/genus level. (**E**) LEfSe analysis—taxonomic cladogram. (**F**) LEfSe analysis—LDA bar chart. Red arrows indicate the microorganisms shared between LEfSe analysis and random forest analysis.

At the phylum level, taxa with an average abundance of >5% in each group were regarded as the dominant phyla (Figure 5A). The dominant phyla in the CK group were Fusobacteriota (30.79%), Firmicutes_A (30.07%), Proteobacteria (10.12%), and Cyanobacteria (15.14%). In the 6% group, the dominant phyla were Fusobacteriota (39.24%), Firmicutes_A (7.18%), Proteobacteria (16.91%), Cyanobacteria (10.39%), Actinobacteriota (7.26%), and Bacteroidota (7.61%). In the 12% group, Fusobacteriota (15.24%), Firmicutes_A (12.90%), Proteobacteria (23.07%), Cyanobacteria (24.28%), Actinobacteriota (7.01%), and Firmicutes_D (6.55%) were dominant. The most common phyla in the CK, 6%, and 12% groups were Fusobacterium, Firmicutes_A, Proteobacteria, and Cyanobacteria, respectively. The phylum most shared between the 6% and 12% groups was Actinobacteria. Bacteroidetes was unique to the 6% group and Firmicutes_D to the 12% group.

At the genus level, taxa with an average abundance of >5% in each group were regarded as the dominant genera (Figure 5C). The dominant genera in the CK group were *Cetobacterium_A* (30.61%) and NIES-981 (10.06%). In the 6% group, they were *Cetobacterium_A* (39.07%), NIES-981 (6.43%), and *Paludibacter* (5.18%). In the 12% group, they were *Cetobacterium_A* (14.40%), NIES-981 (13.51%), and *Aeromonas* (7.27%). The dominant genera shared among the CK, 6%, and 12% groups were *Cetobacterium_A* and NIES-981; *Paludibacter* was only found in the 6% group, and *Aeromonas* only occurred in the 12% group.

We used linear discriminant analysis effect size (LEfSe) (Figure 5E,F) and random forest analyses (Figure 5B,D) to identify biomarker species with significant differences among the groups. Microbes that were ranked in the top three in LEfSe analysis and those that appeared in the results of both analyses were considered biomarkers. In the LEfSe analysis, screening was performed with a linear discriminant analysis (LDA) score of three as the cutoff. Based on these criteria, the biomarker taxa were as follows: 6% group, genus *Paludibacter*; 12% group, genus *Cyanobium_A*, phylum Dependentiae; *Hyphomicrobium_A*, phylum Patescibacteria; *Rhabdaerophilum*. The dominant genus *Paludibacter* (LDA > 4) was considered an important biomarker species for the 6% group, as were *Cyanobium_A* (LDA > 4) and *Rhabdaerophilum*, belonging to the dominant phylum Proteobacteria in the 12% group.

### 3.8. Prediction Analysis of Biological Functions of Intestinal Microbes

Functional prediction of the microbiota in each group was performed using PICRUSt2 analysis and the KEGG database (Supplementary Figure S7). The functions of each group (CK, 6%, and 12%) were mainly enriched in metabolic pathways, with a high abundance of amino acids (4213.51, 4653.2, 4596.14), carbohydrates (4903.08, 5324.1, 4971.1), cofactors and vitamins (4965.97, 5294.36, 4954.43), and terpenoids and polyketides (4101.75, 4160.96, and 3755.98). The abundance of the lipid metabolism pathway in the CK group (2300.72) was lower than that in the 6% (2420.94) and 12% (2477.53) groups.

## 4. Discussion

The liver is the central organ of lipid metabolism in animals and regulates serum TG and TC levels, affecting LDL-C levels [26,27]. Here, replacing soybean meal with ultrafine pulverized mulberry leaf combined with multi-enzyme preparations in grass carp diets reduced serum TG, TC, and LDL-C levels. This is consistent with the findings of previous studies indicating that mulberry leaf or its extract reduces hepatic lipid accumulation [12,28,29]. Mulberry leaf extract reduces abdominal fat deposition in broiler chickens and significantly decreases hepatic lipid accumulation in mice [6,30]. Therefore, replacing soybean meal with ultrafine pulverized mulberry leaves combined with multi-enzyme preparations may reduce fat deposition via multiple pathways.

Serum HDL-C levels decreased in all the mulberry leaf groups, which is inconsistent with previous studies in mice and laying hens that reported increased HDL-C levels [31,32]. We suggest the following possible reasons. Consistent with previous reports on Chinese giant salamander (*Andrias davidianus*) and crucian carp (*Carassius auratus gibelio*) [33,34], our results showed that mulberry leaves can enhance intestinal lipase activity. Enhanced lipase activity implies that more fat-degrading products may be produced. However, the intestines of the experimental fish were at a developmental stage, with limited digestive and absorptive capacity. Therefore, incompletely absorbed fat degradation products in the intestine were converted into short-chain fatty acids (SCFAs) by the microbiota, which inhibited hepatic cholesterol synthesis and reduced serum LDL-C levels [35]. As a result, serum TC decreases, and the amount of cholesterol that needs to be transported back to the liver is also reduced. This may explain the decreased serum HDL-C levels observed in this study [36]. This corroborates the lipid-lowering effect of replacing soybean meal with ultrafine pulverized mulberry leaves combined with multi-enzyme preparations.

Lipid oxidation is central to maintaining energy homeostasis and metabolic health, and the antioxidant capacity of the body is closely linked to lipid metabolism [37,38]. Here, the levels of MDA, a marker of lipid oxidation, increased in the 6% group but decreased in the 12% group. We hypothesized that the increase in the 6% group may have resulted from the abnormal accumulation of large amounts of fatty acids and TG in the hepatocyte cytoplasm when hepatic metabolism was impaired. This led to extensive lipid peroxidation and MDA production [39]. Ultrafine pulverization can improve the extraction efficiency of active components from mulberry leaves and enhance their antioxidant activities [40]. The higher content of bioactive components in the 12% group may have reduced the MDA levels. In this study, the activities of GSH-Px and SOD, and T-AOC were lower in the mulberry leaf groups than in the CK group. This result is similar to reports on mulberry leaf flavonoids fed to water buffaloes [41] but differs from some studies showing that in Xiangdong black goats and laying hens, mulberry leaves regulate the antioxidant status of animals by increasing serum antioxidant enzyme activities and T-AOC [42,43]. Following the lipid-lowering effect of mulberry leaves, the reduced lipid substrates may have alleviated oxidative stress, and the decreases in these parameters reflect an adaptive downregulation of the antioxidant defense system rather than an impairment.

Muscle pH and water-holding capacity are intuitive indicators of meat quality, with higher values indicating better quality [44]. Compared to the CK group, the 6% and 12% groups showed significantly increased muscle pH and significantly reduced CL. Meanwhile, the 12% group also had significantly lower muscle shear force. Reduced CL indicates enhanced water-holding capacity, which is associated with reduced lipid oxidation and increased cell wall integrity [45]. An increase in muscle pH is linked to glucose metabolism [46]. A higher pH favors calpain activity, which improves postmortem muscle tenderness by degrading muscle proteins, which is consistent with the tenderness improvement reflected by the reduced shear force. Similarly, studies on Chinese hybrid pigs [9] and crossbred black goats [47] have shown that mulberry leaves reduce muscle shear force, and that mulberry leaf flavonoids may be the main active components responsible. Here, replacing soybean meal with ultrafine pulverized mulberry leaves combined with multi-enzyme preparations improved the quality of the dorsal muscle of grass carp, indirectly supporting its lipid-lowering effects.

As one of the three major organic nutrients, lipids provide energy to animals and affect muscle flavor, tenderness, and nutritional value, with fatty acid composition being a key factor [48]. Fatty acids are classified into saturated fatty acids (SFAs) and UFAs based on the presence of double bonds in the carbon chain; the latter includes MUFA and PUFA. In this study, muscle EE content decreased with increasing substitution levels of ultrafine pulverized mulberry leaves combined with multi-enzyme preparations. SFA decreased only in the 6% group, whereas UFA decreased in all mulberry leaf groups. The MUFA content increased in the mulberry leaf groups, whereas PUFA showed the opposite trend. Except for PUFA, changes in fatty acids and muscle EE content were consistent with the results reported for broilers fed mulberry leaf extract [6]. This may be due to species differences and the form of mulberry leaves, that is, whole leaf vs. extract. UFA are the main source of muscle flavor, and a moderate increase in their content can improve meat flavor and nutritional value. However, excessive levels increase the rate of muscle oxidation [47]. PUFA are prone to peroxidation, which can damage cell membranes and lead to cell death [49,50]. Replacing soybean meal with ultrafine pulverized mulberry leaves combined with multi-enzyme preparations can reduce fat deposition in the dorsal muscle of grass carp and improve the fatty acid composition. The PUFA content remained significantly lower in the mulberry leaf treatment groups than in the CK group throughout the experiment. This suggests that the lipid-lowering effect of mulberry leaves may reduce substrates for oxidative stress.

The liver is the central site of glycolipid metabolism and the expression levels of key metabolic genes reflect the activity of these pathways [51,52]. In terms of glucose metabolism, downregulation of genes encoding rate-limiting enzymes of gluconeogenesis (*g6pc*, *pepck*) and upregulation of key glycolytic enzyme genes (*gk*, *pfk*, *pk*) generally help reduce blood glucose and improve glucose tolerance [53]. The blood glucose-lowering effect of mulberry leaf or its active components in rodents is associated with the upregulation of proteins encoded by *gk*, *pk*, and *pfk* and the downregulation of those encoded by *g6pc* and *pepck* [54,55]. Here, the relative expression of *g6pc* in the 12% group was lower than that in the CK group. Meanwhile, the relative expression of *pepck* in the 6% group was higher than that in the CK group. This indicates that different gluconeogenic genes respond differently to the replacement of soybean meal with ultrafine pulverized mulberry leaves combined with multi-enzyme preparations. However, in the mulberry leaf group, the expression of *gk*, *pfk*, and *pk* was downregulated, suggesting that this treatment inhibited hepatic glycolysis. In terms of lipid metabolism, the mulberry leaf extract can reduce hepatic TC and abdominal fat deposition, which are associated with the inhibition of FAS and ACC expression [6,30]. A study on largemouth bass showed that mulberry leaf oligosaccharides could inhibit the expression of *acc* and *fas* genes [56]. A similar finding was observed here, in which the expression of *fas* and *acc* was significantly downregulated in the mulberry leaf groups. This indicated that lipogenesis was inhibited in the mulberry leaf groups. Given that glycolysis produces acetyl-CoA for *de novo* fatty acid synthesis [57], we propose that the lipid-lowering effect of replacing soybean meal with ultrafine pulverized mulberry leaves combined with multi-enzyme preparations is associated with the inhibition of hepatic glycolysis and lipogenesis.

Multiple signaling molecules produced by the gut microbiota and intestinal lipid transport regulate lipid homeostasis by affecting host lipid metabolism, hepatic bile secretion, reverse cholesterol transport, and insulin sensitivity [58,59]. Multiple differential metabolites, namely phthalic anhydride, 4-formylsalicylic acid, di-N-propylamine, and monoisobutyl phthalic acid, related to lipid metabolism changed significantly after replacing soybean meal with ultrafine pulverized mulberry leaves combined with multi-enzyme preparations. Among them, the abundance of phthalic anhydride, 4-formylsalicylic acid, di-N-propylamine, and monoisobutyl phthalic acid was significantly reduced in the mulberry leaf groups. Except for di-N-propylamine, which was significantly lower in the 12% group than in the 6% group, the other metabolites showed higher abundances in the 12% group. As a precursor of phthalate esters, phthalic anhydride and its homologue, dibutyl phthalate, can disrupt microbiota homeostasis via the gut–liver axis, induce the upregulation of hepatic lipid synthesis genes, increase serum TC and TG levels, lead to hepatic lipid metabolism disorders and liver inflammation, and inhibit AMPK phosphorylation to hinder lipolysis [60,61]. Mono-(2-ethylhexyl) phthalate, an active metabolite of phthalate esters, induces adipogenesis, promotes fat deposition, and increases serum TG and TC [62]. Monoisobutyl phthalic acid was positively correlated with phthalic anhydride, suggesting that they may synergistically affect lipid metabolism. 4-Formylsalicylic acid is a salicylic acid derivative and sodium salicylate, a metabolite of salicylic acid, likely promotes fat use and reduces plasma fatty acids by activating AMPK [63]. This may indirectly participate in the regulation of lipid metabolism. Di-N-propylamine showed an inconsistent trend with the other metabolites in this study and no literature has clearly linked it to lipid metabolism. Therefore, its mechanism of action requires further investigation. KEGG analysis showed that the differential metabolites in all comparison groups were mainly enriched in the lysine degradation pathway, with the common differential metabolite alpha-ketoadipic acid being downregulated in the mulberry leaf groups. As an intermediate of lysine degradation, alpha-ketoadipic acid can be metabolized to synthesize acetyl-CoA [64], which is a core biosynthetic precursor for fatty acid synthesis [65]. Downregulation of alpha-ketoadipic acid may indicate that fatty acid synthesis and lysine degradation are inhibited in the host. In broilers, lysine deficiency can inhibit the expression of lipid synthesis-related genes and reduce fat deposition [66]. A study on ginseng polysaccharides reported that hepatic lipid accumulation could be reduced by regulating fecal microbiota and the lysine degradation pathway [67]. Therefore, replacing soybean meal with ultrafine pulverized mulberry leaves combined with multi-enzyme preparations may reduce lipogenesis by inhibiting the lysine degradation pathway, with alpha-ketoadipic acid being the key downregulated metabolite. These differential metabolites were positively correlated with lipid metabolism indicators. Phthalic anhydride, 4-formylsalicylic acid, di-N-propylamine, monoisobutyl phthalic acid, and alpha-ketoadipic acid were the key differential metabolites in the intestine that affected lipid metabolism, and the lysine degradation pathway was the core regulatory pathway.

Intestinal microbiota has a characteristic community structure that protects the host from pathogenic infections and contributes significantly to nutrient metabolism [68,69]. Although the beta diversity analysis showed an overall similarity in community structure among the groups, the alpha diversity and Venn diagrams showed significant changes in species composition and many unique species in each group. Therefore, replacing soybean meal with ultrafine pulverized mulberry leaves combined with multi-enzyme preparations promotes internal restructuring rather than reshaping the microbial community. The biomarker species and dominant genera in each group belonged to several phyla, namely, Fusobacteria (Cetobacterium_A), Firmicutes_A (Paludibacter), Proteobacteria (Rhabdaerophilum), and Cyanobacteria (Cyanobium_A; NIES-981). This was consistent with the rank–abundance curve results, which showed that the grass carp intestinal community was dominated by several phyla. However, their abundance did not differ significantly among the groups. This indicates that the treatment effect manifested as the fine-tuning of multiple microbes rather than significant changes in a single flora.

Based on the metabolomic results identifying lysine degradation as a key pathway, we focused on amino acid metabolism in the functional prediction results. The abundance of amino acids increased in the 6% and 12% mulberry leaf groups, reflecting an enhanced microbial capacity to perform this function. Amino acids are exchanged bidirectionally between the host and intestinal microbes [70], and high amino acid levels can upregulate hepatic fatty acid synthesis genes [71]. Enhanced microbial degradation of amino acids can prevent their conversion to fat storage. Instead, amino acids are converted to SCFAs [72,73]. After absorption, SCFAs inhibit hepatic lipogenesis and reduce fat deposition by regulating hormone and gene expression [74,75,76,77,78,79]. The gut microbiota of the mulberry leaf groups exhibited stronger lipid metabolic capacity. Intestinal microbes consume dietary fat and primary bile acids synthesized from cholesterol and secreted by the host [80]. These bile acids promote lipid absorption and act as hormone-like molecules that regulate glycolipid metabolism [81]. Microbial metabolites of lipids and other nutrients also affect host lipid metabolism [82,83]. Therefore, the synergistic enhancement of microbial capacity for amino acid and lipid metabolism may reduce the host lipid supply at the source by enhancing SCFA production and competitively consuming lipids and bile acids, inhibiting lipogenesis and deposition.

The improvement in multiple key indicators exhibited a clear dose-dependent effect, with the best results observed in the 12% group. These changes are consistent with the trends reported in previous studies, such as the dose-dependent decrease in muscle SFA content in black goats with increasing mulberry leaf supplementation [42] and the linear reduction in abdominal fat deposition in broilers with mulberry leaf extract [6]. These findings indicate that adding powdered leaves to feed inhibits fat deposition. The 12% group converted the non-significant change in muscle shear force in the 6% group into a significant reduction, that is, 29.74% lower than that in the CK group, and reduced the elevated hepatic MDA levels observed in the 6% group, that is, 14.43% lower than that in the CK group. This is consistent with reports that mulberry leaf flavonoids improve meat quality [9] and that mulberry leaf extract enhances antioxidant capacity [43].

## 5. Conclusions

These results suggest that replacing soybean meal with ultrafine pulverized mulberry leaves combined with multi-enzyme preparations may influence hepatic glycolipid metabolism through gut microbial metabolism, regulating systemic lipid metabolism, reducing fat deposition, and altering fatty acid composition in grass carp. The reduction in fat deposition, changes in specific fatty acid profiles, and alterations in antioxidant indices suggest that lipid-lowering effects could alleviate oxidative stress. Our findings confirm the lipid-lowering effects of replacing soybean meal with ultrafine pulverized mulberry leaf combined with multi-enzyme preparations in grass carp and its potential mechanisms and further suggest a link between lipid reduction and oxidative stress. This provides a theoretical basis and practical guidance for the precise application of mulberry leaf as a functional feed additive in healthy grass carp aquaculture.

The 12% group showed a better overall effect in regulating lipid metabolism, improving meat quality, and alleviating oxidative stress. It is recommended that a 12% dietary proportion of ultrafine pulverized mulberry leaf combined with multi-enzyme preparations be used to replace soybean meal in grass carp feed. However, this dietary substitution did not significantly affect grass carp growth.

## Figures and Tables

**Table 1:** Composition and nutrient levels of the diet (air-dried basis, %).

Items	Content		
	CK	6%	12%
Ingredients			
Ultrafine pulverized mulberry leaf	0.00	6.00	12.00
Cottonseed meal	6.20	3.43	0.65
Soybean meal	30.60	24.60	18.60
Casein	6.33	9.46	12.60
Cellulose	3.94	3.73	3.52
Distiller dried grains with solubles	4.00	4.00	4.00
Fish meal	12.60	12.60	12.60
Corn starch	7.00	7.00	7.00
Soybean oil	4.83	4.68	4.53
Ca(H_2_PO_4_)_2_	2.00	2.00	2.00
C_5_H_14_NOCl	0.50	0.50	0.50
Wheat flour	21.00	21.00	21.00
Vitamin and mineral premix ^(1)^	0.90	0.90	0.90
Multi-enzyme preparations	0.00	0.10	0.10
Zeolite powder	0.10	0.00	0.00
Total	100.00	100.00	100.00
Nutrient levels ^(2)^			
Crude protein (CP)	33.05	33.12	32.97
Ether extract (EE)	5.40	5.14	5.34
Crude Fiber (CF)	7.15	6.86	6.57
Ash	5.86	6.09	6.32
Phosphorus (P)	1.25	1.21	1.16
Lysine (Lys)	2.00	2.00	2.00
Gross Energy (GE, kJ/g)	16.48	16.50	16.51

Note: ^(1)^ The vitamin premix provides the following per kg of diet: vitamin B_1_ 20.00 mg, riboflavin 20.00 mg, vitamin B_6_ 12.00 mg, vitamin B_12_ 0.15 mg, vitamin K_3_ 12.00 mg, inositol 300.00 mg, pantothenic acid 60.00 mg, niacin acid 70.00 mg, folic acid 10.00 mg, biotin 1.00 mg, vitamin A 8000.00 IU, vitamin D_3_ 2000.00 IU, vitamin E 100.00 mg, vitamin C 500.00 mg, ethoxyquin 200.00 mg, defatted rice bran 535.00 mg; The mineral premix provides the following per kg of diet: KCl 250.00 mg, KI (1.00%) 80.00 mg, CoCl_2_·6H_2_O (1.00%) 70.00 mg, CuSO_4_·5H_2_O 40.00 mg, FeSO_4_·H_2_O 500.00 mg, ZnSO_4_·H_2_O 500.00 mg, MnSO_4_·H_2_O 200.00 mg, Na_2_SeO_3_·5H_2_O (1.00%) 70.00 mg, MgSO_4_·H_2_O 2500.00 mg, zeolite power 3790.00 mg. ^(2)^ CP, EE, and GE were measured values, while the other nutrient levels were calculated values.

**Table 2:** Primer sequence.

Gene	Primer Sequence (5′–3′)		Tm (°C)	bp	GenBank
*gk*	Forward	GGACAATGGTGAACTGGAGGACTTC	59.9	139	GU065314
	Reverse	TAGCAGCACAAGACGCACAAGC	61.0		
*pfk*	Forward	AGAGGAGGGTGTTTGTGGTG	57.4	233	KP262352.1
	Reverse	ATGGATGAAATCGGTGGTGT	53.8		
*pk*	Forward	GCCGAGAAAGTCTTCATCGCACAG	60.8	157	JQ951928
	Reverse	CGTCCAGAACCGCATTAGCCAC	61.5		
*g6pc*	Forward	GTCTCCTCGGTGGCGGAT	60.6	150	XM_051914690.1
	Reverse	CTCACCGAAAAGCACCCACT	58.1		
*pepck*	Forward	AGGAAGATCGTGTCGTTCGG	57.2	138	XM_051897832.1
	Reverse	AGTGATGCCCAGAATCAGCA	56.6		
*cpt1*	Forward	GCATCCATGACACGTTTATTC	51.5	118	JF728839
	Reverse	GAAGTTTCTCTTCTCTCGTCTC	52.2		
*lpl*	Forward	CCGCAAAAACCAGAGATTGC	55.1	80	XM_051909470.1
	Reverse	TCAACGTGCGCCTTTACTCT	56.8		
*ppar-ɑ*	Forward	CGCTGAGGTTCGGATATTT	52.0	112	FJ623265
	Reverse	ACGTCACCTGGTCATTTAAG	51.9		
*fas*	Forward	AGGTCGCCTTCCTGAGTCTA	57.6	114	XM_051913957.1
	Reverse	GGAAGGCCATACAGACCTGG	58.3		
*acc*	Forward	GGCACAAAGACCGACAGATCA	57.7	122	XM_051892870.1
	Reverse	GGCCTGGAAGCGTTTAGACT	57.7		
*β-actin*	Forward	TTACCACTTCACGCCGACTC	57.5	119	XM_051886219.1
	Reverse	GTCACCTTCACCGTTCCAGT	57.5		

**Table 3:** Growth performance of grass carp (*n* = 4).

Items	Groups		
	CK	6%	12%
Initial body weight (g)	8.42 ± 0.14	8.43 ± 0.15	8.43 ± 0.18
Final body weight (g)	82.69 ± 3.96	75.01 ± 1.90	75.89 ± 5.17
Feed intake (g)	83.01 ± 0.26	83.01 ± 0.26	83.28 ± 0.53
Weight gain rate (%)	881.84 ± 47.05	790.17 ± 22.52	800.30 ± 61.29
Specific growth rate (%/d)	3.79 ± 0.08	3.64 ± 0.04	3.63 ± 0.11
Feed conversion ratio	1.16 ± 0.07	1.25 ± 0.03	1.29 ± 0.10
Survival rate (%)	99.38 ± 0.63	99.38 ± 0.63	98.75 ± 1.25
Hepatosomatic index (%)	1.97 ± 0.25 ^a^	0.99 ± 0.12 ^b^	1.23 ± 0.12 ^b^

^a, b^ Different superscripts in the same row indicate significant differences between groups (*p* < 0.05).

**Table 4:** Serum indicators and intestinal lipase activity of grass carp (*n* = 4).

Items	Groups		
	CK	6%	12%
TG (mmol/L)	1.67 ± 0.00 ^a^	1.61 ± 0.00 ^b^	1.53 ± 0.00 ^c^
TC (mmol/L)	5.24 ± 0.08 ^a^	4.12 ± 0.04 ^b^	4.16 ± 0.02 ^b^
HDL-C (mmol/L)	3.14 ± 0.05 ^a^	2.72 ± 0.02 ^b^	2.68 ± 0.01 ^b^
LDL-C (mmol/L)	2.09 ± 0.10 ^a^	1.40 ± 0.02 ^b^	1.45 ± 0.01 ^b^
GSH-Px (nmol/min/mL)	84.64 ± 0.64 ^a^	59.36 ± 0.55 ^b^	59.55 ± 1.02 ^b^
T-AOC (U/mL)	13.53 ± 0.23 ^a^	13.47 ± 0.36 ^a^	10.82 ± 0.17 ^b^
MDA (nmol/mL)	2.01 ± 0.04 ^b^	2.29 ± 0.04 ^a^	1.72 ± 0.03 ^c^
SOD (U/mL)	53.27 ± 1.35 ^a^	43.70 ± 1.18 ^b^	41.16 ± 0.92 ^b^
Lipase (U/g)	1.66 ± 0.03 ^c^	2.49 ± 0.04 ^b^	8.80 ± 0.10 ^a^

^a, b, c^ Different superscripts in the same row indicate significant differences between groups (*p* < 0.05).

**Table 5:** Physicochemical properties and routine nutritional components of back muscle (*n* = 4).

Items	Groups		
	CK	6%	12%
pH	6.67 ± 0.06 ^b^	7.20 ± 0.04 ^a^	7.14 ± 0.07 ^a^
Meat Color (%)	81.55 ± 1.71 ^ab^	84.60 ± 1.10 ^a^	75.73 ± 2.57 ^b^
Shear Force (N)	17.55 ± 1.37 ^a^	16.98 ± 0.67 ^a^	12.33 ± 0.58 ^b^
Cooking Loss (%)	70.64 ± 4.57	71.64 ± 3.41	76.28 ± 1.61
Centrifugal Loss (%)	21.97 ± 0.69 ^b^	12.33 ± 1.77 ^a^	12.17 ± 1.59 ^a^
CP (g/100 g)	16.78 ± 0.29 ^ab^	17.13 ± 0.14 ^a^	16.50 ± 0.09 ^b^
EE (g/100 g)	4.33 ± 0.08 ^a^	3.18 ± 0.03 ^b^	2.80 ± 0.04 ^c^
Ash (g/100 g)	2.38 ± 0.03	2.48 ± 0.05	2.48 ± 0.03

^a, b, c^ Different superscripts in the same row indicate significant differences between groups (*p* < 0.05).

**Table 6:** Fatty acid composition of dorsal muscle (mg/kg, *n* = 4).

Items	Groups		
	CK	6%	12%
SFA	123.76 ± 0.35 ^a^	112.41 ± 0.30 ^b^	123.93 ± 0.64 ^a^
Palmitic acid C16:0	59.75 ± 0.27 ^b^	58.33 ± 0.20 ^c^	61.27 ± 0.54 ^a^
Tricosanoic acid C23:0	11.91 ± 0.03 ^c^	12.75 ± 0.25 ^b^	18.59 ± 0.21 ^a^
UFA	48.45 ± 0.85 ^a^	35.15 ± 0.12 ^c^	39.50 ± 0.09 ^b^
MUFA	4.44 ± 0.08 ^c^	5.75 ± 0.07 ^a^	4.81 ± 0.03 ^b^
Oleic acid C18:1n9c	0.45 ± 0.01 ^c^	0.72 ± 0.01 ^a^	0.69 ± 0.00 ^b^
PUFA	44.01 ± 0.82 ^a^	29.40 ± 0.15 ^c^	34.69 ± 0.08 ^b^
ω-3 PUFA	6.89 ± 0.04 ^a^	4.66 ± 0.04 ^c^	4.90 ± 0.03 ^b^
EPA C20:5n3	2.00 ± 0.02 ^a^	0.96 ± 0.03 ^c^	1.77 ± 0.03 ^b^
DHA C22:6n3	4.31 ± 0.03 ^a^	3.00 ± 0.02 ^b^	2.69 ± 0.03 ^c^
ω-6 PUFA	37.12 ± 0.80 ^a^	24.75 ± 0.14 ^c^	29.80 ± 0.08 ^b^
Linoleic acid C18:2n6c	17.95 ± 0.22 ^a^	12.16 ± 0.19 ^b^	12.41 ± 0.18 ^b^

Eicosapentaenoic acid, EPA; Docosahexaenoic acid, DHA; ^a, b, c^ Different superscripts in the same row indicate significant differences between groups (*p* < 0.05).

## Data Availability

Data will be made available from the corresponding author on request. Intestinal microbial sequencing data can be found at: https://www.ncbi.nlm.nih.gov/bioproject/?term=PRJNA1418311 (accessed on 13 October 2026). Intestinal Non-targeted Metabolomics sequencing data can be found at: https://www.ebi.ac.uk/metabolights/MTBLS13847 (accessed on 13 October 2026).

## Supplementary Materials

The following supporting information can be downloaded at https://www.mdpi.com/article/10.3390/antiox15101350/s1, Figure S1: Quality control analysis (*n* = 4). (A,B) QC mass spectrum alignment plots (POS, NEG). The *x*-axis represents the retention time of each chromatographic peak, and the *y*-axis represents the peak intensity. (C,D) QC correlation analysis (POS, NEG). The x- and y-axes represent individual QC samples. Each point in the grid represents an ion peak (metabolite) extracted from the QC samples, and the x- and y-coordinates represent the log-transformed signal intensity of the ion peaks. The values shown are correlation coefficients. (E,F) QC relative standard deviation (POS, NEG). The *x*-axis represents the RSD value, and the *y*-axis represents the proportion of peaks relative to the total number of peaks in the QC samples. (G,H) Overall PCA analysis (POS, NEG). PC1 represents principal component 1, PC2 represents principal component 2, each point represents one sample, and points of different colors indicate different groups; Figure S2: Multivariate statistical PLS-DA analysis (*n* = 4). (A,B) CK vs. 6%; (C,D) CK vs. 12%; (E,F) 6% vs. 12%; (G,H) CK vs. 6% vs. 12%. A, C, E, and G are positive ion mode; B, D, F, and H are negative ion mode. PC1 represents principal component 1, PC2 represents principal component 2, each point represents one sample, and points of different colors indicate different groups; Figure S3: Univariate statistical analysis (*n* = 4). (A,B) CK vs. 6%; (C,D) CK vs. 12%; (E,F) 6% vs. 12%. A, C, and E are positive ion mode; B, D, and F are negative ion mode. The *x*-axis represents the log2 value of the fold change, and the *y*-axis represents the -log10 value of the significance *p* value. Significantly different metabolites: metabolites with FC > 2 and *p* value < 0.05 are shown in red, metabolites with FC < 0.5 and *p* value < 0.05 are shown in blue, and non-significant metabolites are shown in gray; Figure S4: Screening of differential metabolites (*n* = 4). (A) Positive ion mode; (B) Negative ion mode; (C) Combined positive and negative ion mode. The *x*-axis represents the comparison group, and the *y*-axis represents the number of differential metabolites. Red indicates upregulation, and blue indicates downregulation; Figure S5: Machine learning analysis of differential metabolites (*n* = 4). (A) CK vs. 6%; (B) CK vs. 12%; (C) 6% vs. 12%; (D) CK vs. 6% vs. 12%. In the bar charts, the *x*-axis represents the importance score of each species for the classifier model, and the *y*-axis represents the metabolite name. The heatmap shows the abundance distribution of these metabolites across samples. From top to bottom, the importance of metabolites to the model decreases successively. Red arrows indicate common differential metabolites among the comparison groups; Figure S6: Correlation analysis of differential metabolites. Both the vertical and oblique vertical axes represent the names of differential metabolites. The color represents the correlation, with red indicating positive correlation and blue indicating negative correlation; the darker the color, the stronger the correlation. Red arrows indicate the screened differential metabolites; Figure S7: Functional potential prediction (*n* = 4). (A) CK; (B) 6%; (C) 12%. In the figure, the *x*-axis represents the abundance of functional pathways, and the *y*-axis represents the functional pathways at the second KEGG classification level. The rightmost column indicates the first-level classification to which each pathway belongs. Data shown represent the mean abundance of all selected samples; Table S1: Tables of differential metabolite enrichment analysis. PathwayID: pathway ID; Pathway: pathway name; Up/Down: number of up-/down-regulated metabolites enriched in the pathway; DEM: total number of differential metabolites enriched in the pathway; Total: total number of metabolites in the pathway; Up/Down_meta: IDs (names) of the up-/down-regulated metabolites enriched in the pathway.

## Abbreviations

The following abbreviations are used in this manuscript:

TG	Triglyceride
TC	Total cholesterol
HDL-C	High-density lipoprotein cholesterol
LDL-C	Low-density lipoprotein cholesterol
EE	Ether extract
FI	Feed intake
WGR	Weight gain rate
SGR	Specific growth rate
FCR	Feed conversion ratio
SR	Survival rate
HSI	Hepatosomatic index
T-AOC	Total antioxidant capacity
MDA	Malondialdehyde
SOD	Superoxide dismutase
GSH-Px	Glutathione peroxidase
CP	Crude protein
CL	Centrifugal loss
qPCR	Quantitative real-time PCR
SEM	Standard error of the mean
ANOVA	One-way analysis of variance
UFA	Unsaturated fatty acid
PUFA	Polyunsaturated fatty acid
MUFA	Monounsaturated fatty acid
ASVs	Amplicon sequence variants
OTUs	Operational taxonomic units
LDA	Linear discriminant analysis
SCFAs	Short-chain fatty acids
SFA	Saturated fatty acids
