# Supplementary material for: Replacing Soybean Meal with Ultrafine Pulverized Mulberry Leaf (Combined with Multi-Enzyme Preparations) Reduces Fat Deposition and Alleviates Oxidative Stress in Grass Carp via Gut–Liver Axis Regulation
Source: Antioxidants (Basel). 2026 Oct 19;15(10):1350. doi: 10.3390/antiox15101350 (PMC13701587; doi:10.3390/antiox15101350)
Supplement: Supplementary file 1 [file antioxidants-15-01350-s001.zip › Analysis workflow for muscle fatty acids, gut microbiota, and intestinal non-targeted metabolomics.pdf]

1.1. Gas Chromatography

Take 1 mL of a 37-component fatty acid methyl ester mixed standard, dissolve in n-hexane, and make up to 10 mL to prepare a standard stock solution (1.0 mg/mL), which is stored sealed at 0–4 °C. Pipette the standard stock solution and dilute with n-hexane to prepare a series of standard working solutions with concentrations of 2.5 μg/mL, 5 μg/mL, 10 μg/mL, 50 μg/mL, and 200 μg/mL, prepared fresh before use.

Accurately weigh 0.1–1 g of sample and transferred to a 250 mL flat-bottom flask. Add approximately 100 mg of pyrogallic acid, a few boiling chips, 2 mL of 95% ethanol, and 4 mL of deionized water, then mix well. Subsequently, 5 mL of hydrochloric acid solution was added and mixed again. Place the flask in a 70–80 °C water bath for hydrolysis for 40 min. The flask was shaken every 10 min to mix the particles adhered to the flask wall with the solution. After the hydrolysis, the flasks were cooled to room temperature.

To the hydrolyzed sample, add 5 mL of 95% ethanol and mixed thoroughly. The hydrolysate was transferred from the flask to a separatory funnel, rinsed the flask and stopper with 25 mL of petroleum ether, and rinsed again into a separatory funnel. Cap the funnel, shake for 5 min, and allow to stand for 10 min. The ether layer was extracted into a 250 mL flask. Repeat the extraction of the hydrolysate 3 times as described above, and the separatory funnel was rinsed with a petroleum ether–diethyl ether mixture. All rinses were collected in a 250 mL flask. The residue was concentrated to dryness by using a rotary evaporator.

Add 4 mL of 2% sodium hydroxide–methanol solution to the fat extract, connect a reflux condenser, and reflux in an 80 ± 1 °C water bath until oil droplets disappear. Add 3.5 mL of 15% boron trifluoride–methanol solution from the top of the reflux condenser, and continue refluxing in an 80 ± 1 °C water bath for 2 min. The reflux condenser was rinsed with a small amount of water. The heating was stopped, the flask was removed from the water bath, and cooled rapidly to room temperature.

Accurately add 7.5 mL of n-heptane was added and shaken for 2 min, and a saturated aqueous sodium chloride solution was added and allowed to stand for layer separation. Pipette approximately 2.5 mL of the upper n-heptane extract into a 25 mL test tube. Approximately 2 g of anhydrous sodium sulfate was added, shaken for 1 min, allowed to stand for 5 min, and the upper solution was pipetted into a sample vial for analysis.

Chromatographic conditions: Nitrogen (carrier gas); CD-2560 capillary column (100 m × 0.25 mm × 0.2 μm); column temperature: 140 °C held for 5 min, then increased to 240 °C at 1 °C/min and held for 30 min; injection temperature: 250 °C; split ratio: 1:10.

1.2. Microbiome Analysis

(1) DNA Extraction: Total genomic DNA samples were extracted using the OMEGA Soil DNA Kit (M5635-02) (Omega Bio-Tek, Norcross, GA, USA), following the manufacturer's instructions, and stored at-20°C prior to further analysis. The quantity and quality of extracted DNAs were measured using a NanoDrop NC2000 spectrophotometer (Thermo Fisher Scientific, Waltham, MA, USA) and agarose gel electrophoresis, respectively.

(2) 16S rRNAGene Amplicon Sequencing: PCR amplification of the bacterial 16S rRNA genes V3–V4 region was performed using the forward primer 338F (5'-ACTCCTACGGGAGGCAGCA-3') and the reverse primer 806R (5'-GGACTACHVGGGTWTCTAAT-3'). Sample-specific 7-bp barcodes were incorporated into the primers for multiplex sequencing. The PCR components contained 5 μl of buffer (5×), 0.25 μl of Fast pfu DNA Polymerase (5U/μl), 2 μl (2.5 mM) of dNTPs, 1 μl (10 μM) of each Forward and Reverse primer, 1 μl of DNA Template, and 14.75 μl of ddH2O. Thermal cycling consisted of initial denaturation at 98 °C for 5 min, followed by 25 cycles consisting of denaturation at 98

°C for 30 s, annealing at 53 °C for 30 s, and extension at 72 °C for 45 s, with a final extension of 5 min at 72 °C. The PCR amplicons were purified using Vazyme VAHTSTM DNA Clean Beads (Vazyme, Nanjing, China) and quantified using a Quant-iT PicoGreen dsDNA Assay Kit (Invitrogen, Carlsbad, CA, USA). After the individual quantification step, the amplicons were pooled in equal amounts, and paired-end 2×250 bp sequencing was performed using the Illumina NovaSeq platform with the NovaSeq 6000 SP Reagent Kit (500 cycles) at Shanghai Personal Biotechnology Co., Ltd (Shanghai, China).

(3) Sequence Analysis: Sequence data were obtained using QIIME2 2022.11(Bolyen et al., 2019) with slight modifications according to the official tutorials (https://docs.qiime2.org/2022.11/tutorials/).

(4) Bioinformatics and statistical analyses: Analyses were performed using the Personal Genes Cloud Platform (https://www.genescloud.cn/home).

1.3. Non-targeted Metabolomics Analysis

(1) Metabolite extraction: Weigh 50 mg of sample into a 2 mL centrifuge tube; Add 200 μL of pre-cooled water, add 2 steel beads, put into a high-throughput tissue grinder, 55 Hz, grind for 60 s, repeat this step once; Add 800 μL methanol : acetonitrile (1:1, v/v); Place in ultrasonic cleaning machine for 30 min; Freeze in a-20 ℃ refrigerator for 30 min; Centrifuge at 12,000 rpm and 4°C for 10 min, take 800 μL of the supernatant and concentrate to dryness under vacuum; Add 150 μL 50 % methanol (containing 5 ppm 2-chlorophenylalanine) to dissolve and vortex for 30 seconds; Centrifuge at 12,000 rpm and 4°C for 10 min, filter the supernatant through a 0.22 μm filter membrane, and add the filtrate to the test bottle; Take 10-20 μL of each sample filtrate and mix them into a QC sample for evaluating instrument stability and data reliability

(2) Chromatographic methods: An ACQUITY UPLCHSST3column (100Å, 1.8 μm, 2.1 mm × 100 mm) was used with a flow rate of 0.4 mL/min, a column temperature of 40°C, an autosampler temperature of 8°C, and an injection volume of 2 μL. Positive and negative mode mobile phase: mobile phase A is 0.1 % formic acid water, mobile phase B is acetonitrile (containing 0.1 % formic acid), and the elution gradient is as follows: Time (0, 1, 7, 8, 8.1, 12 min) and corresponding B % (5, 5, 95, 95, 5, 5 %).

(3) Mass spectrometry: A Thermo Orbitrap Exploris 120 mass spectrometer was used to collect DDA mass spectrometric data in positive and negative ion modes using Xcalibur software (version: 4.7, Thermo). HESI source, spray voltage 3.5 kV/-3.0 kV, sheath gas 40 arb, auxiliary gas 15 arb, capillary temperature 325 ℃, auxiliary gas temperature 300 ℃, primary resolution 60,000, scan range 100-1000 m/z, AGC Target Standard, Max IT 100 ms, the top 4 ions were screened for secondary fragmentation, dynamic exclusion time was 8 s, secondary resolution 15,000, HCD collision energy 30 %, AGC Target Standard, Max IT Auto. All formal and QC samples were loaded into the equipment according to the aforementioned chromatography and mass spectrometry methods. Before formal injection, the QC samples were injected to 2-4 times to balance the system. During the injection process, one QC sample was injected every 5-10 samples for subsequent data evaluation and quality control.

(4) Metabolite library search: Raw data were imported into the commercial software Compound Discoverer™ 3.3 (version 3.3.2.31, Thermo, Waltham, USA). Peak extraction, alignment, correction, and other operations were performed based on the new peak detection and quality scoring algorithms of the software.

(5) Bioinformatics and statistical analyses: Analyses were performed using the Personal Genes Cloud Platform (https://www.genescloud.cn/home).
