# Supplementary material for: Replacing Soybean Meal with Ultrafine Pulverized Mulberry Leaf (Combined with Multi-Enzyme Preparations) Reduces Fat Deposition and Alleviates Oxidative Stress in Grass Carp via Gut–Liver Axis Regulation
Source: Antioxidants (Basel). 2026 Oct 19;15(10):1350. doi: 10.3390/antiox15101350 (PMC13701587; doi:10.3390/antiox15101350)
Supplement: Supplementary file 1 [file antioxidants-15-01350-s001.zip › Supplementary Figures.pdf]

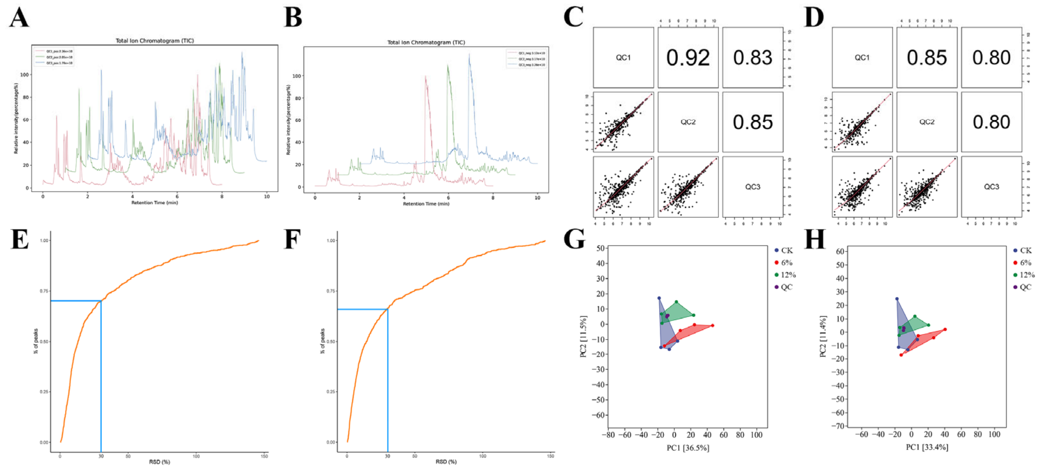

Figure S1. Quality control analysis (n = 4). (A, B) QC mass spectrum alignment plots (POS, NEG). The x-axis represents the retention time of each chromatographic peak, and the y-axis represents the peak intensity. (C, D) QC correlation analysis (POS, NEG). The x- and y-axes represent individual QC samples. Each point in the grid represents an ion peak (metabolite) extracted from the QC samples, and the x- and y-coordinates represent the log-transformed signal intensity of the ion peaks. The values shown are correlation coefficients. (E, F) QC relative standard deviation (POS, NEG). The x-axis represents the RSD value, and the y-axis represents the proportion of peaks relative to the total number of peaks in the QC samples. (G, H) Overall PCA analysis (POS, NEG). PC1 represents principal component 1, PC2 represents principal component 2, each point represents one sample, and points of different colors indicate different groups.

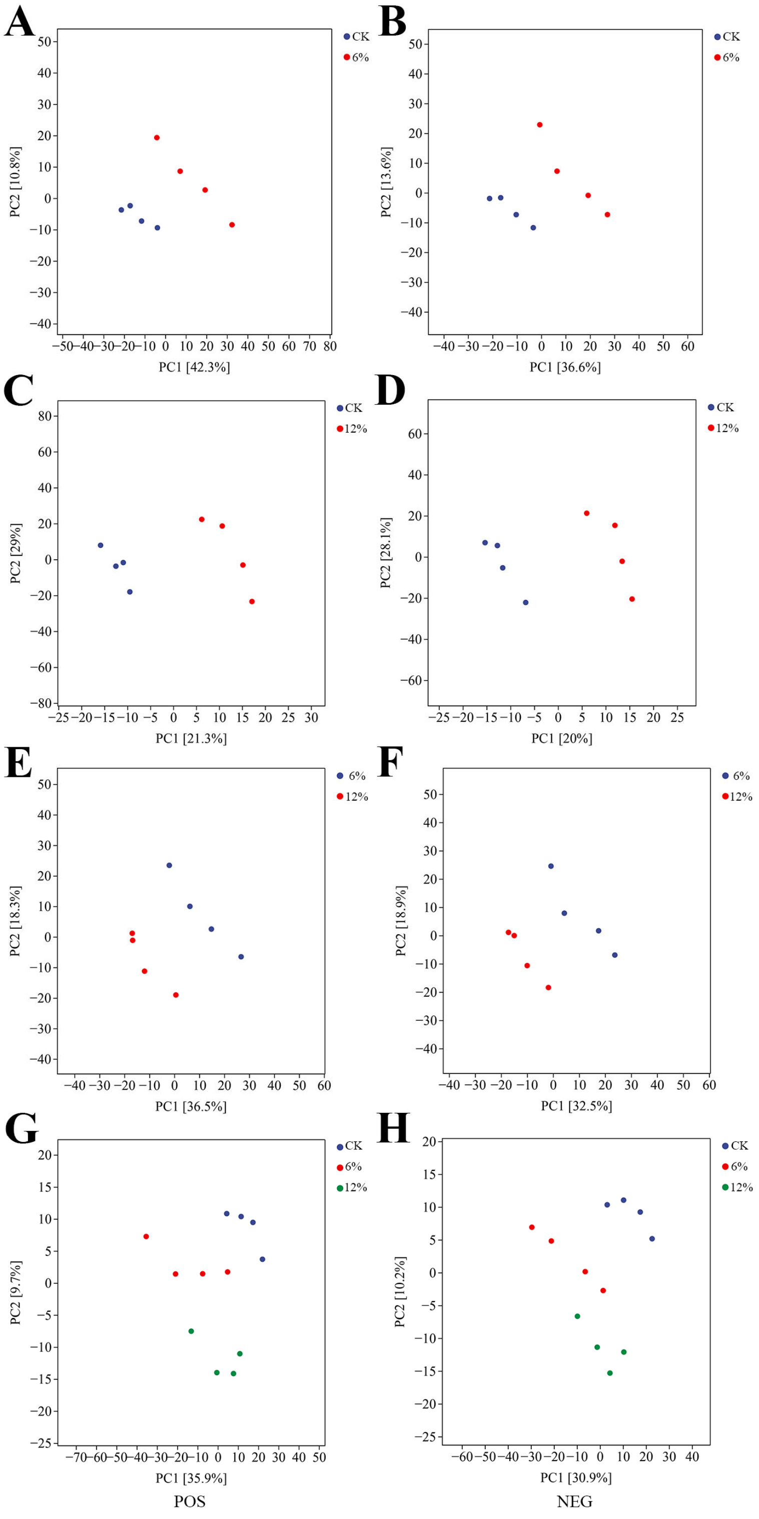

Figure S2. Multivariate statistical PLS-DA analysis (n = 4). (A, B) CK vs 6%; (C, D) CK vs 12%; (E, F) 6% vs 12%; (G, H) CK vs 6% vs 12%. A, C, E, and G are positive ion mode; B, D, F, and H are negative ion mode. PC1 represents principal component 1, PC2 represents principal component 2, each point represents one sample, and points of different colors indicate different groups.

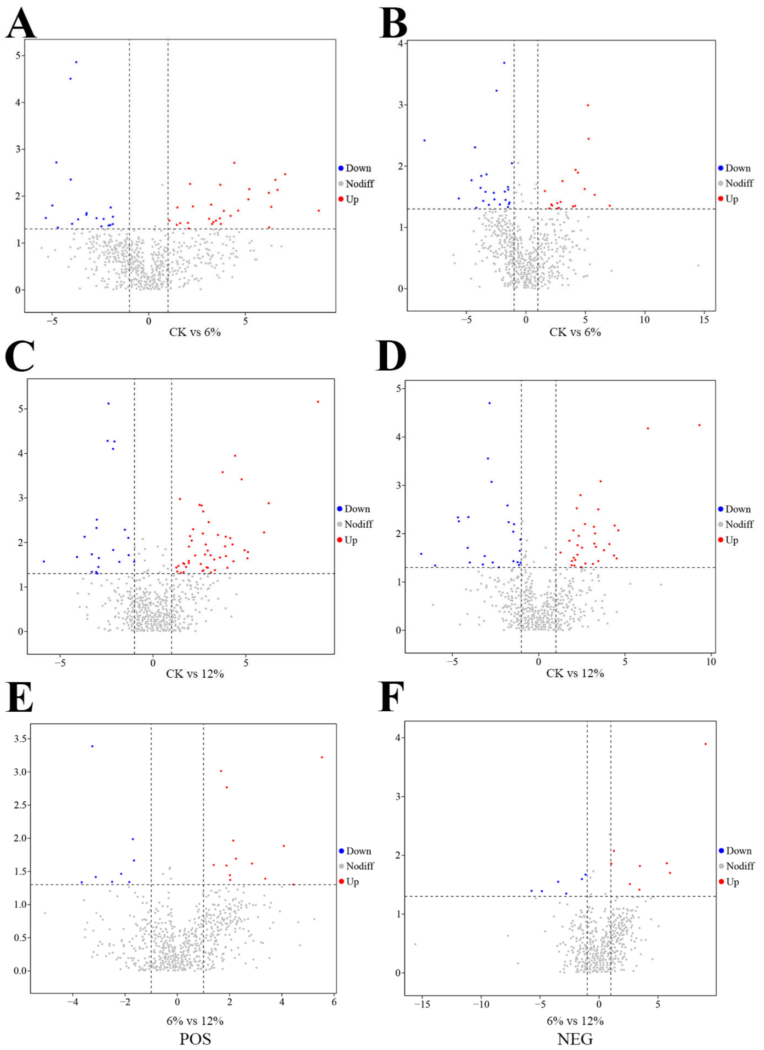

Figure S3. Univariate statistical analysis (n = 4). (A, B) CK vs 6%; (C, D) CK vs 12%; (E, F) 6% vs 12%. A, C, and E are positive ion mode; B, D, and F are negative ion mode. The x-axis represents the log2 value of the fold change, and the y-axis represents the -log10 value of the significance P value. Significantly different metabolites: metabolites with FC > 2 and *P* value < 0.05 are shown in red, metabolites with FC < 0.5 and *P* value < 0.05 are shown in blue, and non-significant metabolites are shown in gray.

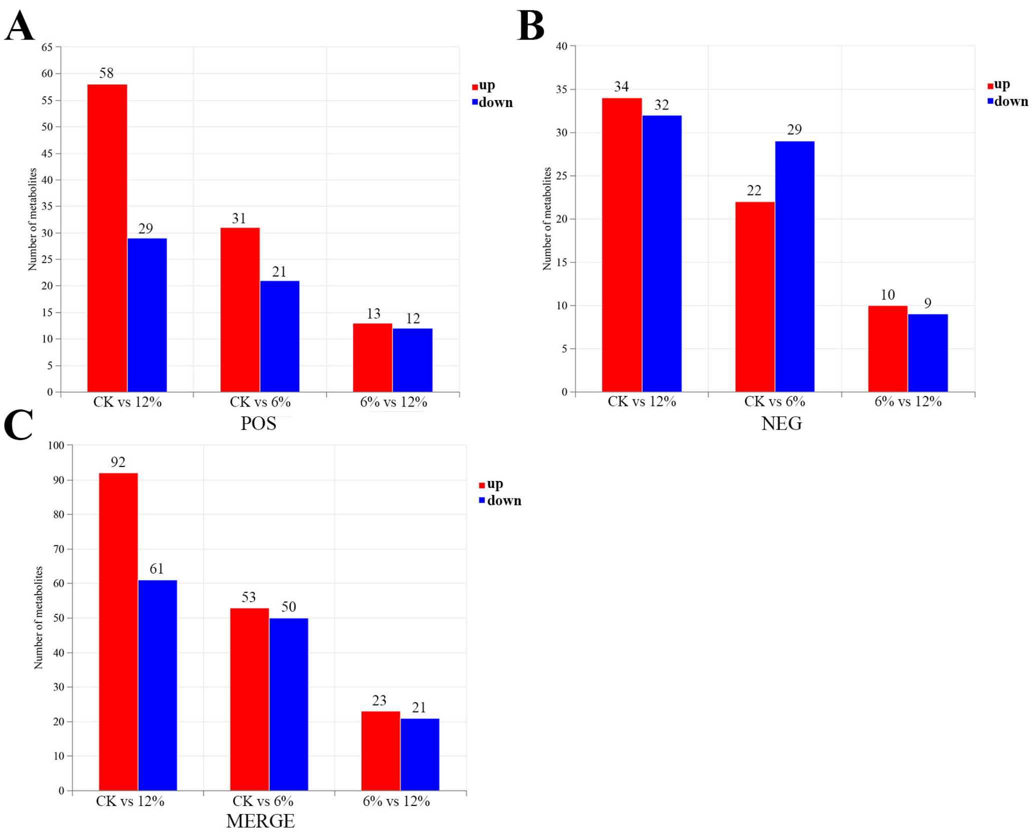

Figure S4. Screening of differential metabolites (n = 4). (A) Positive ion mode; (B) Negative ion mode; (C) Combined positive and negative ion mode. The x-axis represents the comparison group, and the y-axis represents the number of differential metabolites. Red indicates upregulation, and blue indicates downregulation.

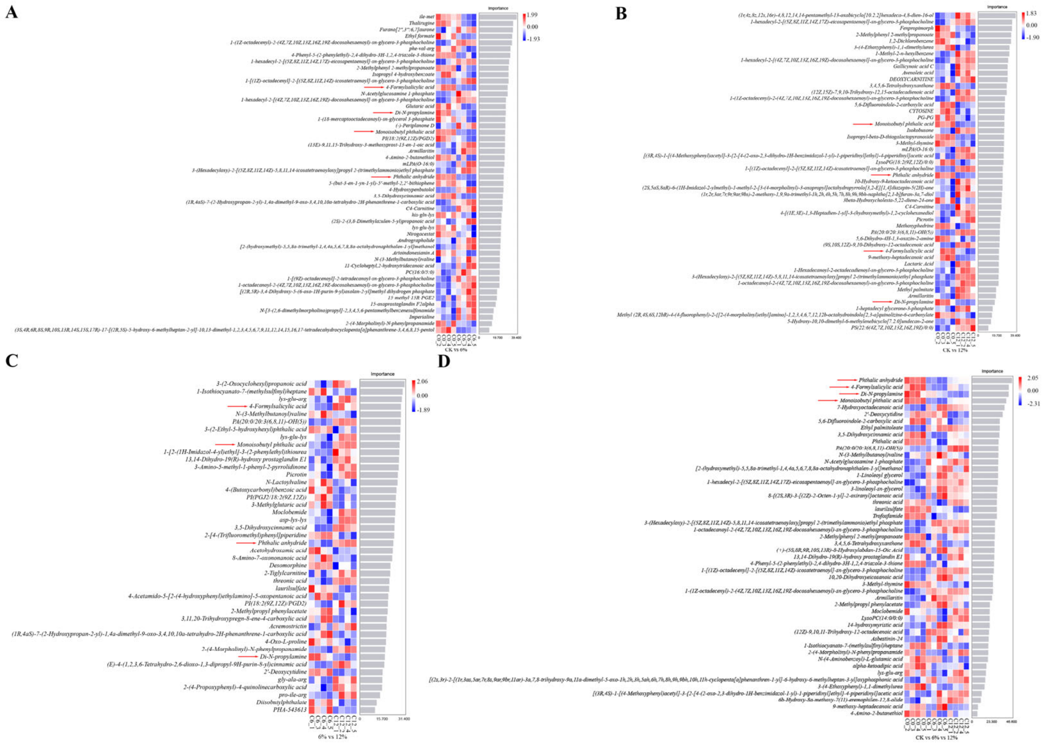

Figure S5. Machine learning analysis of differential metabolites (n = 4). (A) CK vs 6%; (B) CK vs 12%; (C) 6% vs 12%; (D) CK vs 6% vs 12%. In the bar charts, the x-axis represents the importance score of each species for the classifier model, and the y-axis represents the metabolite name. The heatmap shows the abundance distribution of these metabolites across samples. From top to bottom, the importance of metabolites to the model decreases successively. Red arrows indicate common differential metabolites among the comparison groups.

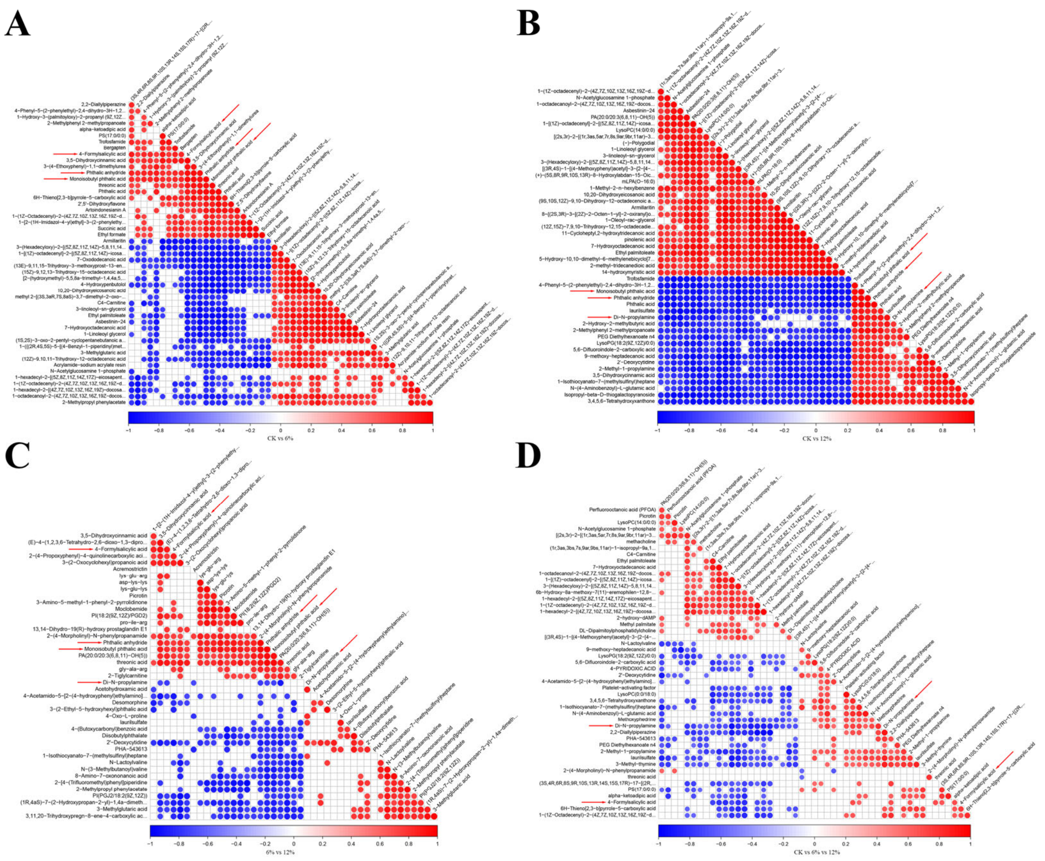

Figure S6. Correlation analysis of differential metabolites. Both the vertical and oblique vertical axes represent the names of differential metabolites. The color represents the correlation, with red indicating positive correlation and blue indicating negative correlation; the darker the color, the stronger the correlation. Red arrows indicate the screened differential metabolites.

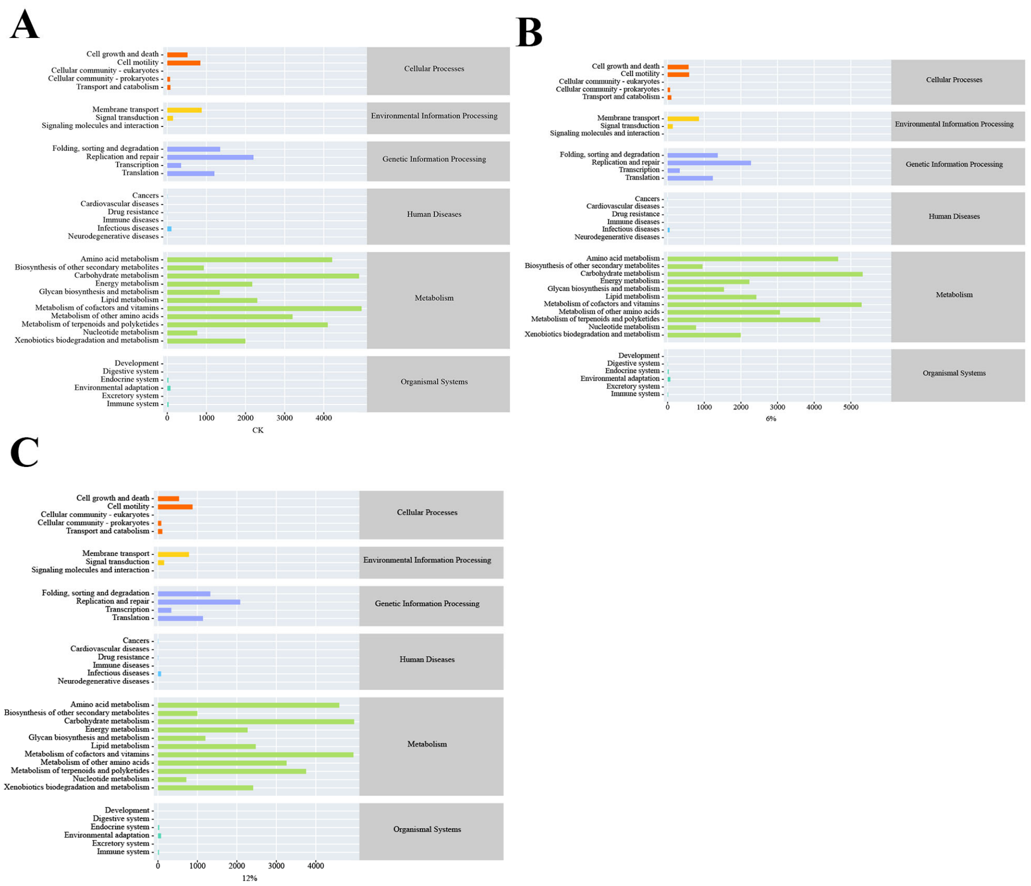

Figure S7. Functional potential prediction (n = 4). (A) CK; (B) 6%; (C) 12%. In the figure, the x-axis represents the abundance of functional pathways, and the y-axis represents the functional pathways at the second KEGG classification level. The rightmost column indicates the first-level classification to which each pathway belongs. Data shown represent the mean abundance of all selected samples.
